# Periprostatic adipocytes act as a driving force for prostate cancer progression in obesity

**DOI:** 10.1038/ncomms10230

**Published:** 2016-01-12

**Authors:** Victor Laurent, Adrien Guérard, Catherine Mazerolles, Sophie Le Gonidec, Aurélie Toulet, Laurence Nieto, Falek Zaidi, Bilal Majed, David Garandeau, Youri Socrier, Muriel Golzio, Thomas Cadoudal, Karima Chaoui, Cedric Dray, Bernard Monsarrat, Odile Schiltz, Yuan Yuan Wang, Bettina Couderc, Philippe Valet, Bernard Malavaud, Catherine Muller

**Affiliations:** 1 Université de Toulouse, UPS, Toulouse F-31077, France; 2 Département “Biologie du Cancer” et “Biologie Structurale et Biophysique”, CNRS; Institut de Pharmacologie et de Biologie Structurale, Toulouse F-31077, France; 3 Département d'Anatomo-Pathologie, Institut Universitaire du Cancer, Toulouse cedex 9 31059, France; 4 Département “Tissu Adipeux, Obésité et Diabète”, Institut National de la Santé et de la Recherche Médicale, INSERM U1048, Toulouse F-31432, France; 5 Centre Hospitalier de la Région de Saint-Omer (CHRSO), Route de Blendecques, BP 60357, Saint-Omer Cedex 62505, France; 6 Département “Tumeur et Environnement”, Centre de Recherche en Cancérologie de Toulouse (CRCT), Toulouse Cedex 1 F-31037, France; 7 Département d'Urologie, Institut Universitaire du Cancer, Toulouse cedex 9 31059, France

## Abstract

Obesity favours the occurrence of locally disseminated prostate cancer in the periprostatic adipose tissue (PPAT) surrounding the prostate gland. Here we show that adipocytes from PPAT support the directed migration of prostate cancer cells and that this event is strongly promoted by obesity. This process is dependent on the secretion of the chemokine CCL7 by adipocytes, which diffuses from PPAT to the peripheral zone of the prostate, stimulating the migration of CCR3 expressing tumour cells. In obesity, higher secretion of CCL7 by adipocytes facilitates extraprostatic extension. The observed increase in migration associated with obesity is totally abrogated when the CCR3/CCL7 axis is inhibited. In human prostate cancer tumours, expression of the CCR3 receptor is associated with the occurrence of aggressive disease with extended local dissemination and a higher risk of biochemical recurrence, highlighting the potential benefit of CCR3 antagonists in the treatment of prostate cancer.

## Abstract (web summary)

Obesity is associated with an elevated risk of prostate cancer. Here, the authors show that periprostatic adipose tissue promotes the migration and local invasion of prostate cancer cells by secreting the chemokine, CCL7, and that this process is enhanced in the context of obesity.

## Graphical abstract

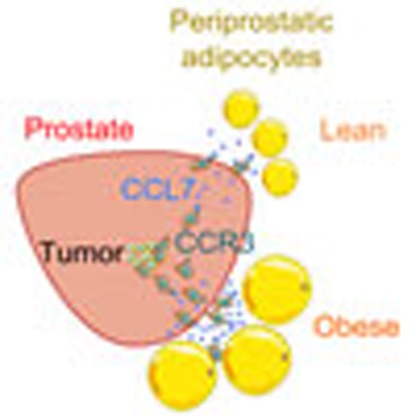

Extraprostatic extension is a widely acknowledged adverse factor in prostate cancer^1^, and an important determinant of prostate cancer recurrence after treatment^2^. The prostate gland is surrounded by periprostatic adipose tissue (PPAT), which is, like other fat depots, an active endocrine organ. Adipose tissue is mainly comprised of adipocytes, although other cell types contained in the so-called stromal vascular fraction (SVF) contribute to its growth and function, including adipocyte-derived stem cells, preadipocytes, lymphocytes, macrophages, fibroblasts and vascular endothelial cells^3^,^4^. Mature adipocytes, first thought of as energy-storing cells, have emerged this last decade as highly endocrine cells, which are able to secrete hormones, growth factors, chemokines or pro-inflammatory molecules, an heterogeneous group of molecules termed ‘adipokines'^3^,^4^.

It is increasingly clear that obesity, where the normal balance of adipose tissue secretory proteins is perturbed, is associated with a greater risk of aggressive prostate cancer with increased local dissemination^5^,^6^. Obesity, in particular excess visceral adiposity, leads to changes in the cellular composition of adipose tissue (mainly infiltration by macrophages) as well as to modifications of the secretory pattern of mature adipocytes^3^,^4^. The existing correlation between the abundance of PPAT and tumour aggressiveness suggests a paracrine role for this fat depot during tumorigenesis^7^. Limited data exists on the mechanisms that could be involved in this effect. We uncovered that the secretions of mature adipocytes possess a strong ability to support the directed migration of prostate cancer cells, suggesting that mature adipocytes can affect the early stages of prostate cancer progression by promoting the spread of cancer cells outside the prostate gland. This switch from a prostate-confined tumour to a locally disseminated cancer is viewed as a crucial step in the progression of the disease at a clinical level^2^.

A complex network of chemokines, and their associated receptors, influences the directed migration of invasive cancer cells^8^. Chemokines comprise a large group of small secreted proteins (8–11 kDa in size) that are grouped into four families (C, CC, CXC and CX3C) depending on the spacing of key cysteine residues near their N terminus, with the CC and CXC families representing the bulk of known chemokines^8^. Directed migration of cells that express the appropriate chemokine receptor occurs along a chemokine gradient, allowing cells to move towards high local concentrations of ligand, a process known as chemotaxis. The functions of chemokines in malignancy depend considerably on the chemokine type and on tumour- and host-dictated characteristics^8^. Many chemokines and their receptors affect the development and progression of prostate cancer, the most evidence being provided for CCR2, CXCR1, CXCR2 and CXCR4 (ref. 9). Expression of these four chemokine receptors is higher in human prostate cancer tissues when compared with normal epithelium or tissues presenting benign prostatic hyperplasia, and their expression could correlate with tumour aggressiveness^10^,^11^,^12^. More recently, it has been demonstrated that a prostate cancer cell line, Du-145, also expresses the CCR3 receptor^13^, whose over-expression was described previously in human melanoma or kidney cancers^14^,^15^. Chemokines are released by the tumour cells themselves and/or by host cells, including infiltrating leukocytes, endothelial cells and fibroblasts^8^,^9^.

Few studies have investigated the role of mature adipocytes in this context, although these cells secrete chemokines, the production of which is upregulated in obesity^16^. Using combined *in vitro* and *in vivo* approaches, we demonstrate here that the ability of PPAT to attract cancer cells away from the prostate gland is dependent on an original CCR3/CCL7 axis. The upregulation of CCL7 secretion in obesity facilitates extraprostatic extension and increases local dissemination, this effect being totally abrogated when the CCR3/CCL7 axis is inhibited. Our study unravels a new pathway, regulated by obesity, implicating PPAT in prostate cancer aggressiveness and suggests new strategies for treatment of advanced prostate cancer involving CCR3 antagonists, which are currently being developed for other diseases including asthma^17^.

## Results

### CCR3 drives Ad-CM-induced migration of prostate cancer cells

**Figure 1:**
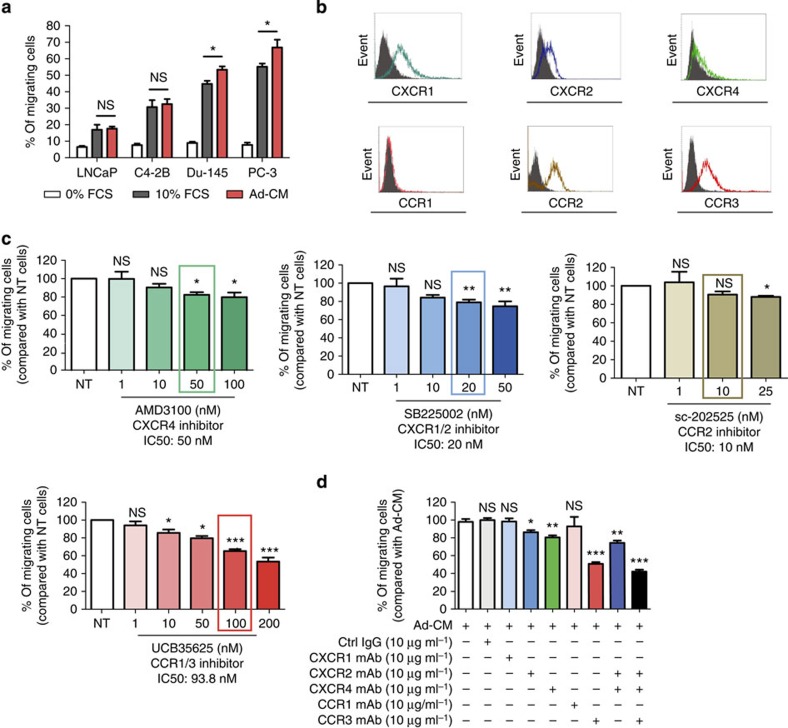
Adipocytes promote the migration of prostate cancer cells in a CCR3-dependent manner. (**a**) *In vitro* migration of the indicated prostate cancer cell lines towards a medium containing either 0% (used as a negative control) or 10% FCS, or towards conditioned medium obtained from 3T3-F442A mature adipocytes (Ad-CM). Data are shown as mean±s.e.m. (*n*=5). (**b**) One representative experiment out of three showing the expression of chemokine receptors (CXCR1, CXCR2, CXCR4, CCR1, CCR2 and CCR3) detected by flow cytometry in the human prostate cancer cell line, PC-3. Solid histogram: control isotypes; open histogram: indicated antigens. (**c**) *In vitro* migration towards Ad-CM in the presence of increasing doses of the indicated receptor antagonists. Cells were pre-incubated with the receptor antagonists for 30 min at 37 °C before their addition to Transwell chambers. The receptor antagonists were also present during the migration assay (12 h). The molecules were used within a range of doses that inhibit chemotaxis; the line surrounding the histogram indicates the previously described IC_50_ of the inhibitors in chemotaxis experiments. Data are shown as mean±s.e.m (*n*=3). (**d**) Similar experiments were performed in the presence of blocking monoclonal antibodies (mAbs) directed against CXCR1, CXCR2, CXCR4, CCR1, CCR3 or control IgG at 10 μg ml^−1^ used alone or in combination. Bar plots represent the percentage of migrating cells relative to the migration of untreated cells (set to 100%). Data are shown as mean±s.e.m (*n*=3). The statistical significance of differences between means of migrating cells (in %) in treated versus untreated (NT) cells was evaluated with Student's *t*-tests. Statistical analysis: * statistically significant by Student's *t*-test *P*<0.05, **P<0.01, ***P<0.001, NS, not significant. *n* stands for the number of replicated independent experiments.

The conditioned medium from 3T3-F442A adipocytes (Ad-CM) significantly promoted the directed migration of all prostate cancer cell lines studied as compared with the negative control using 0% fetal calf serum (FCS). The migration of the most aggressive cell lines, PC-3 and Du-145, was even slightly higher than that observed with 10% FCS (Fig. 1a). As expected, these cells expressed chemokine receptors important for prostate cancer dissemination in humans (CXCR1, CXCR2, CXCR4 and CCR2)^9^ as well as CCR3 whose function in prostate cancer chemotaxis has been recently described *in vitro*^13^ (Fig. 1b and Supplementary Fig. 1a).

Antagonists of these receptors were used to analyse their involvement in Ad-CM-induced migration. The doses used were within the range of IC_50_ (maximal doses between two- and 2.5-fold IC_50_) and without toxicity on prostate cancer cells (Supplementary Fig. 2). The highest doses of CXCR4 (AMD3100 (ref. 18)), CXCR1/CXCR2 (SB225002 (ref. 19)) or CCR2 (sc-202525 (ref. 20)) antagonists inhibited the migration of PC-3 cells by 10–20% (Fig. 1c). For the CCR2 antagonist, this inhibitory effect was only present at maximal doses and not at the described IC_50_ (ref. 20). For the CCR1/CCR3 antagonist UCB35625 (ref. 21), a clear dose-dependent effect was found and this effect was the strongest among all the inhibitors used (45% inhibition at 200 nM compared with control). A CCR3 neutralizing monoclonal antibody (mAb) gave similar results (50% inhibition compared with control) and migration was unaffected by a mAb against CCR1, which is not expressed by prostate cancer cells (Fig. 1b,d). Use of CXCR2 and CXCR4 neutralizing mAbs also confirmed the results obtained with the pharmacological inhibitors with a decrease of PC-3 migration by 15% and 20%, respectively (Fig. 1d). Combination of both CXCR2 and CXCR4 mAbs inhibited the migration of PC-3 cells by 25% (compared with a 50% inhibition in the presence of CCR3 mAb alone), whereas a 60% decrease was observed in the presence of the three blocking mAbs (CCR3, CXCR2 and CXCR4; Fig. 1d). The combined effect of blocking mAbs appears, therefore, rather additive than synergistic. Similar results were obtained with combination of inhibitors. In all cases, the effect of CCR1/3 antagonist alone was greater than the effect of combination of CXCR1/2, CCR2 and CXCR4 antagonists (Supplementary Fig. 3). Experiments using blocking mAbs and pharmacological inhibitors conducted with Du-145 cells confirmed these results (Supplementary Fig. 1b,c). Taken together, these results highlight that CCR3 alone is a master regulator of prostate cancer cells migration towards Ad-CM.

The CCR3 receptor was expressed in cell lines derived from several tumour models (breast, colon, pancreas and melanoma) that come into contact with surrounding adipose tissue during invasion^22^ (Supplementary Fig. 4a). The migration of these cells in response to Ad-CM was correlated with their aggressiveness. In contrast to prostate cancer cells, inhibition of CCR3 had no effect on the Ad-CM-directed migration of all but one cell line (20% inhibition in the breast cancer cell line MDA-MB231; Supplementary Fig. 4b). These results were confirmed using CCR3 blocking mAb (Supplementary Fig. 5). Use of pharmacological inhibitors and blocking mAbs showed that both CXCR2 and CXCR4 are predominantly involved in the migration of the other cancer models towards Ad-CM (Supplementary Fig. 5). For example, the CXCR4 blocking mAb inhibits the migration of melanoma or pancreatic cancer cells towards Ad-CM by 40% (Supplementary Fig. 5). These results highlight that the balance between active receptors and secreted chemokines varies from one tumour type to another, with the preferential and specific involvement of CCR3 in prostate cancer.

### Role of the CCR3/CCL7 axis in adipocytes-induced chemotaxis

**Figure 2:**
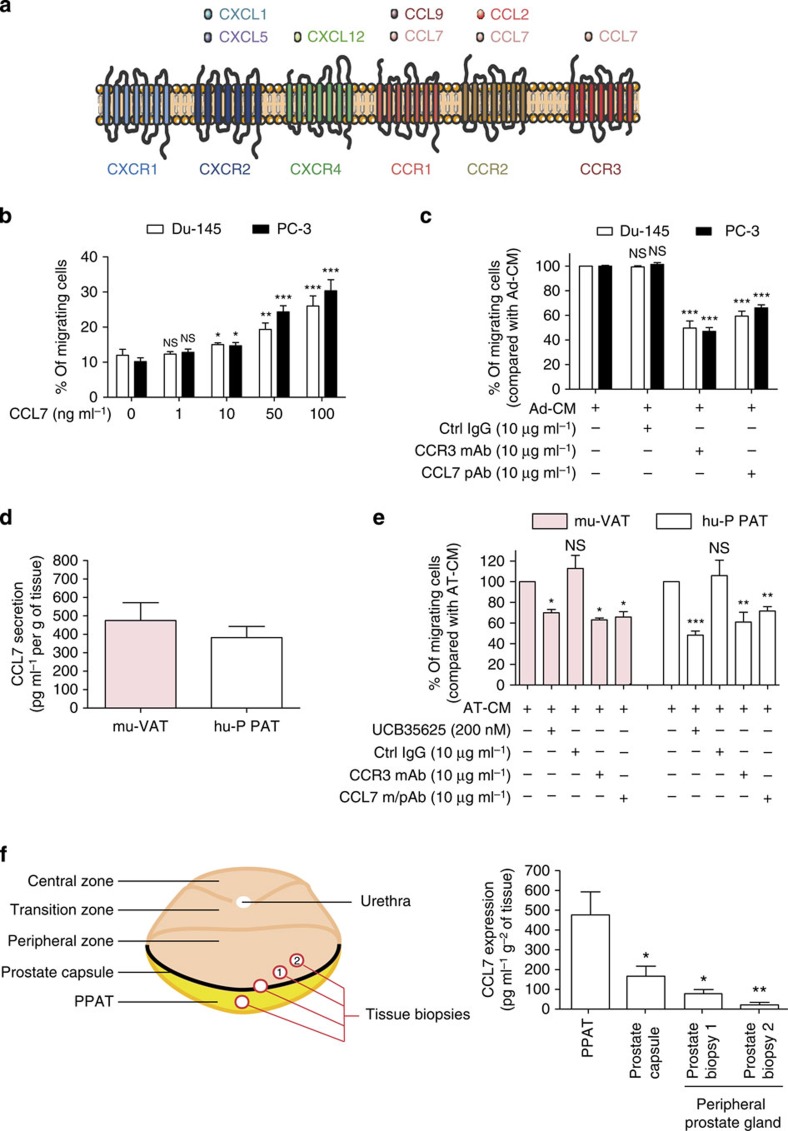
CCL7/CCR3 axis is involved in the directed migration of prostate cancer cells towards PPAT. (**a**) Graphic representation of chemokines identified by proteomic analyses of Ad-CM and their corresponding receptors. (**b**) *In vitro* migration of Du-145 and PC-3 cells towards medium without serum in the presence or absence of CCL7 recombinant protein (1–100 ng ml^−1^). Data are shown as mean±s.e.m. (*n*=3). The statistical differences between the mean percentages of migrating cells in experiments performed in the presence versus in the absence of CCL7 were evaluated with Student's *t*-tests. (**c**) *In vitro* migration of Du-145 and PC-3 cells towards Ad-CM in the presence of m/pAbs directed against CCR3 or CCL7, or control IgG (10 μg ml^−1^). Bar plots represent the percentage of migrating cells relative to the migration of untreated cells (set to 100%). Data are shown as mean±s.e.m (*n*=3). The statistical significance of differences between means of migrating cells (in %) in treated versus untreated cells was evaluated with Student's *t*-tests. (**d**) CCL7 secretion by murine visceral adipose tissue (mu-VAT, three samples) or human periprostatic adipose tissue (hu-PPAT, five samples). Data are shown as mean±s.e.m. (**e**) *In vitro* migration of PC-3 cells towards mu-VAT and hu-PPAT conditioned medium (AT-CM) in the presence or absence of CCR3 antagonist (UCB35625, 200 nM), blocking m/pAbs directed against CCR3 or CCL7, or control IgG (10 μg ml^−1^). Bar plots represent the percentage of migrating cells relative to the migration of untreated cells (set to 100%). Data are shown as mean±s.e.m. (*n*=3). The statistical significance of differences between means of migrating cells (in %) in treated versus untreated cells was evaluated with Student's *t*-tests. (**f**) Graphic representation of the site of the staged biopsies performed in human prostatectomy specimens (*n*=3) is shown (left panel) and the expression of CCL7 in these biopsies is shown (right panel). The mean expression of CCL7 in staged prostate biopsies was compared with the mean expression in PPAT. Statistical analysis: * statistically significant by Student's *t*-test *P*<0.05, **P<0.01, ***P<0.001, NS, not significant. *n* stands for the number of replicated independent experiments. *n* stands for the number of replicated independent experiments.

To detect the CCR3 ligand secreted by adipocytes, we conducted a proteomic analysis of Ad-CM and reproducibly detected six secreted chemokines: CXCL1, CXCL5, CXCL12, CCL2, CCL7 and CCL9, whose presence in Ad-CM was validated by ELISA (Supplementary Table 1). CXCL1 and CXCL5 bind to CXCR2, CXCL12 to CXCR4 and CCL2 to CCR2 (Fig. 2a)^23^. Of note, no ligand for CXCR1 was found in the adipocyte secretome, in accordance with the absence of effect of the CXCR1 blocking mAb on the migration of all the tested models (Fig. 1d, Supplementary Figs 1c and 5). Among the chemokines identified in our proteomic study, only CCL7 has been described as a ligand for CCR3 (ref. 24) (Fig. 2a). Since the secretion of CCL5, another CCR3 ligand, has been described for mammary adipocytes^25^, we dosed this chemokine by ELISA in Ad-CM. As shown in Supplementary Table 1, the level of CCL5 was almost undetectable in Ad-CM (mean concentration 0.01 ng ml^−1^ for 1 × 10^5^ cells compared with 28.3 ng ml^−1^ for CCL7), in accordance with the results of our proteomic analysis. Expression of CCL7 by adipocytes has been only described in one study using the murine pre-adipocyte cell line 3T3-L1 (ref. 26). As illustrated by its alternate name, monocyte chemotactic protein 3 (MCP-3), CCL7 was originally shown to recruit monocytes to sites of injury and mediate local inflammatory responses. This chemokine has since been shown to regulate the migration of multiple immune cells, including T lymphocytes, natural killer cells, immature dendritic cells, basophils and eosinophils^27^. CCL7 could also be implicated in the chemotaxis of cancer cells to metastatic sites, although *in vivo* studies are limited in number and mainly concern gastric and colorectal cancers^28^,^29^. To our knowledge, the role of CCL7 in the chemotaxis of prostate cancer cells has never been studied.

A set of results confirmed the role of CCL7 in the migration of prostate cancer cells towards Ad-CM. Prostate cancer cells (PC-3 and Du-145) underwent chemotaxis in response to increasing doses of recombinant CCL7 (Fig. 2b) and their migration was inhibited by CCL7 neutralizing polyclonal antibody (pAb) when Ad-CM was used as a chemoattractant (Fig. 2c). Similar results were obtained with LNCaP and C4-2B, two less aggressive prostate cancer cell lines, highlighting the role of the CCR3/CCL7 axis in the chemotaxis of prostate cancer cells in response to adipocyte secretions (Supplementary Fig. 6). CCL7 was also detected in CM of mouse visceral adipose tissue (mu-VAT-CM) and in that of human PPAT (hu-PPAT-CM; Fig. 2d). The migration of prostate cancer cells towards CM from these primary tissues also depended on the CCR3/CCL7 axis (Fig. 2e). We sought to assess the role of adipose tissue in the CCL7-directed migration of prostate cancer cells. We first verified that prostate cancer cells do not secrete this chemokine (Supplementary Fig. 7a) and that the inhibition of CCR3/CCL7 did not affect cell migration in response to 10% FCS (Supplementary Fig. 7b). Punch biopsy samples of human prostatectomy specimens taken from the PPAT or from inside the prostate gland revealed a strong gradient of CCL7 expression (Fig. 2f), suggesting that CCL7 is secreted by PPAT and passively diffuses to the prostate peripheral zone through the prostate capsule, that has been shown to be a permeable barrier^30^. Therefore, the gradient of CCL7 found in PPAT will be able to promote the directed migration of invasive tumour cells expressing the CCR3 receptor.

### CCR3/CCL7-induced chemotaxis is enhanced by obesity

**Figure 3:**
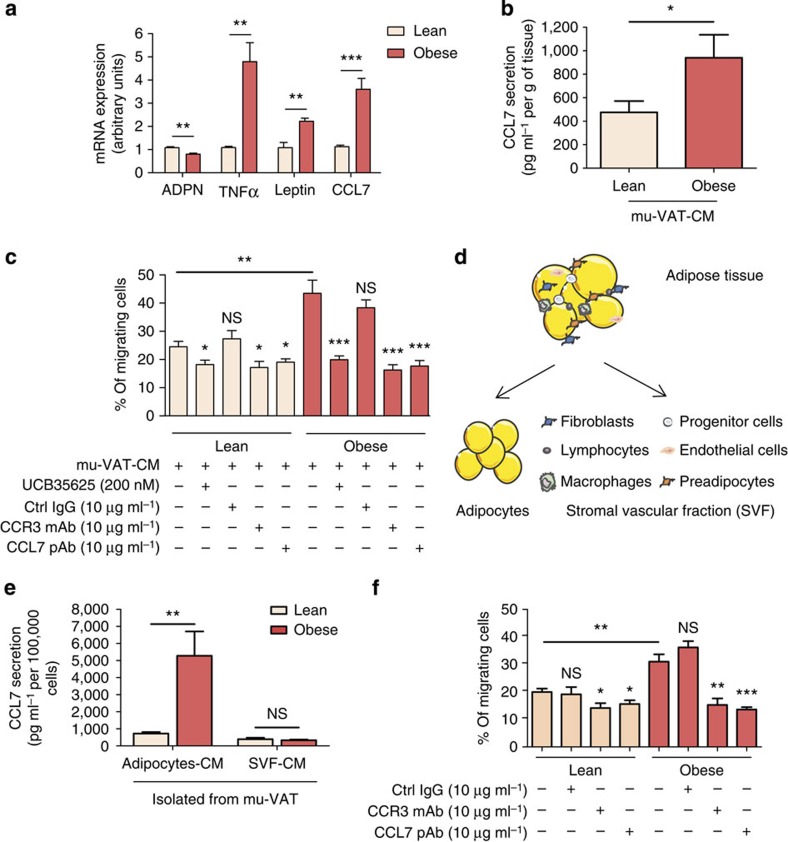
CCL7 secretion by mature adipocytes is positively regulated in obesity and promotes an increased directed migration of prostate cancer cells. (**a**) mRNA were extracted from murine visceral adipose tissue (mu-VAT) of age-matched C57BL/6 mice either lean or obese (after 12 weeks of high-fat diet). Expression of CCL7 mRNA was evaluated by RT-qPCR. A panel of genes whose expression has been shown to decrease (Adiponectin, ADPN) or increase (tumor necrosis factor-α (TNF-α), Leptin) in obesity was used as a control to validate the samples. Data are shown as mean±s.e.m. (*n*=5). (**b**) Secretion of CCL7 in mu-VAT-CM obtained from lean or obese C57BL/6 mice (three animals per group). Data are shown as mean±s.e.m. (*n*=3). (**c**) *In vitro* migration of PC-3 cells towards mu-VAT-CM obtained from lean or obese animals (three animals per group) treated or not with CCR3 inhibitor (UCB35625 200 nM), blocking m/pAbs directed against CCR3 or CCL7, or control isotype (all used at 10 μg ml^−1^). Data are shown as mean±s.e.m. (*n*=3). The statistical significance of differences between means of migrating cells (in %) in treated versus untreated cells was evaluated with Student's *t*-tests. (**d**) Graphic representation of the cellular components of adipose tissue. (**e**) CCL7 secretion by primary adipocytes or SVF cells isolated from mu-VAT of C57BL/6 lean or obese mice (six animals per group). Data are shown as mean±s.e.m. (*n*=3). (**f**) *In vitro* migration of PC-3 cells towards mu-VAT adipocyte-CM. Adipocytes were isolated from the mu-VAT of lean or obese C57BL/6 mice (six animals per group) in the presence or absence of blocking m/pAbs directed against CCR3 or CCL7 or control IgG (10 μg ml^−1^). Data are shown as mean±s.e.m. (*n*=3). The statistical significance of differences between means of migrating cells (in %) in treated versus untreated cells was evaluated with Student's *t*-tests. The differences between the percentages of migrating cells towards Ad-CM isolated from mu-VAT from obese versus lean mice are also shown. Statistical analysis: * statistically significant by Student's *t*-test *P*<0.05, **P<0.01, ***P<0.001, NS, not significant. *n* stands for the number of replicated independent experiments.

Obesity is associated with poor outcome in prostate cancer patients^5^,^6^. In obesity, changes in adipocyte size affect the metabolic and endocrine functions of adipose tissue, and infiltration by macrophages contributes to systemic inflammation and metabolic syndrome. CCL7 has been reported to be over-expressed in adipose tissue in a context of obesity at mRNA levels^16^ and we confirmed this over-expression in the VAT of obese individuals and mice (Fig. 3a and Supplementary Fig. 8a). Leptin and CCL7 expression were correlated, suggesting that CCL7 secretion is related to the hypertrophic state of adipocytes^31^ (Supplementary Fig. 8b). Using ELISA, we confirmed that the secretion of CCL7 was higher in obese, when compared with lean mu-VAT-CM (Fig. 3b).

The migration of prostate cancer cells in response to mu-VAT-CM was significantly higher when cells migrate towards CM obtained from the VAT of obese mice compared with that of lean animals (1.9-fold increase; Fig. 3c). Importantly, this effect was totally abrogated when the CCR3/CCL7 axis was inhibited using pharmacological inhibitors or specific blocking mAbs (Fig. 3c). Mature adipocytes and the so-called SVF were then separated to determine the main source of CCL7 secretion in adipose tissue (Fig. 3d). In lean conditions, the secretion of CCL7 was slightly higher in adipocytes than in the SVF, and a dramatic increase of CCL7 expression was observed in obesity only in mature adipocytes (around fivefold increase compared with lean conditions; Fig. 3e). When the secretions of adipocytes isolated from the mu-VAT of obese mice were used as a chemoattractant, the migration of prostate cancer cells was enhanced compared with secretions from adipocytes isolated from lean mice (Fig. 3f). As shown before for whole adipose tissue, the use of pharmacological inhibitors and CCR3/CCL7 blocking m/pAbs completely abrogated the increase of tumour cell migration observed in obesity (Fig. 3f). The SVF fraction has a slight but significant effect on prostate cancer migration. However, this effect was independent of the CCR3/CCL7 axis and obesity (Supplementary Fig. 9). Adipocytes isolated from human PPAT exhibit very short-term survival in culture (<4 h). To circumvent this problem, we derived adipocytes from *ex vivo* differentiation of progenitors present in the SVF fraction of three patients. These adipocytes secrete CCL7 and use of Ad-CM prepared from these cells supported prostate cancer cells migration in a CCR3-dependent manner (Supplementary Fig. 10). Comparison of cells differentiated from lean versus obese patients would have been informative in the context of our study.

Since it has been demonstrated that mature adipocyte CXCL12 secretion (the ligand for CXCR4) is enhanced by obesity^32^, we investigated the role of the CXCR4/CXCL12 axis in our model. First, we demonstrated that the secretion of CXCL12 in isolated mature adipocytes was only slightly regulated by obesity when compared with CCL7 (1.6-fold increase for CXCL12 and fivefold increase for CCL7; Supplementary Fig. 11a) in accordance with previously published data^32^. Secretion of CXCL12 was also detected at low levels in the SVF, with a slight enhancement in obesity (Supplementary Fig. 11a). Using the conditioned medium from isolated adipocytes, we further demonstrated that both CXCR4 and CXCL12 blocking mAbs inhibit the migration of PC-3 cells by about 20% in obese, but not lean, conditions (Supplementary Fig. 11b). However, the enhanced chemotaxis towards the secretion of mature adipocytes isolated from obese animals was still observed in the presence of CXCR4/CXCL12 blocking mAbs in opposition to CCR3/CCL7 m/pAbs that completely abrogated this effect (Supplementary Fig. 11c). Thus, obesity increases the directed migration of prostate cancer cells by modulating the secretory pattern of mature adipocytes, this effect being mainly dependent on the CCR3/CCL7 axis.

### Over-expression of CCR3 in aggressive human tumours

**Figure 4:**
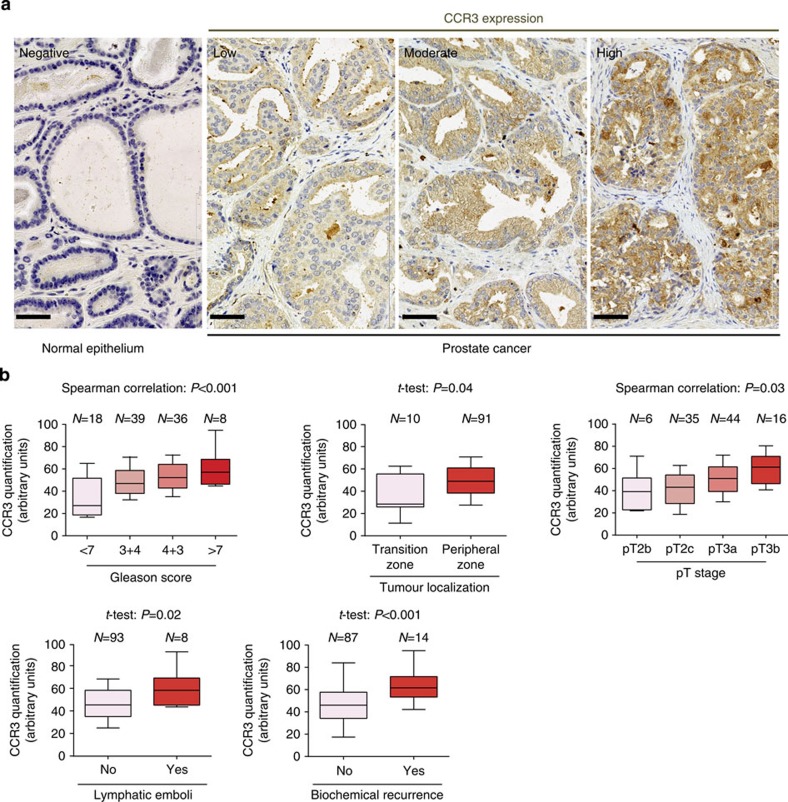
CCR3 is expressed in prostate cancer and its expression is correlated with poor prognosis and a high risk of local extension. (**a**) Immunohistochemical staining for CCR3, as well as hematoxylin counter-staining, was performed in normal epithelium and human prostate cancer tissues. Two pathologists, who were blind to clinical data, independently scored CCR3 expression. Pictures show a representative experiment for each of the staining intensities of CCR3 expression found in human tumours (low, moderate and high expression) whereas normal epithelium remains negative in all tested samples (pictures, zoom 40 × ). Scale bars, 50 μm. (**b**) CCR3 expression was evaluated by immunohistochemistry on a TMA containing 101 tumours in duplicate. The boxes represent the median (black middle line) limited by the 25th (Q1) and 75th (Q3) percentiles. The whiskers are the upper and lower adjacent values, which are the most extreme values within Q3+1.5(Q3−Q1) and Q1−1.5(Q3−Q1), respectively. These box plots represent CCR3 values comparing tumours Gleason score, tumour localization, tumour stage, patients with and without lymphatic emboli or biochemical recurrence. For the correlation between CCR3 expression and variables with more than two ordered classes regarding disease severity (for example, Gleason score or tumour stage), we used Spearman's correlation tests assuming a monotonic relation between considered variables. We used Student's *t*-tests (notified by *) to correlate CCR3 expression to non-ordered categorical variables (for example, presence or not of emboli or biochemical recurrence). The biochemical recurrence, as defined by the European Association of Urology guidelines, corresponded to two PSA readings >0.2 ng ml^−1^.

We sought to investigate the role of CCR3 in human prostate cancer and, to begin with, we studied its expression in a first tissue microarray (TMA) containing 10 normal prostate samples and 91 human prostate cancer annotated with cancer differentiation (Gleason score). CCR3 was expressed in prostate cancer, but not in normal epithelium (Fig. 4a), and its expression was correlated with Gleason score that reflects both the extent of glandular differentiation and the pattern of growth of the tumour in the prostatic stroma (a high score is associated with an aggressive tumour and poor prognosis)^33^ (Supplementary Table 2). Very interestingly, the proportion of tumours expressing moderate and high levels of CCR3 increased in 4+3 compared with 3+4 Gleason scored tumours. Despite a similar global Gleason score (7), it has been shown that a higher contingent of undifferentiated cells within the tumours (Gleason 4+3) significantly worsens the prognosis as compared with tumours that exhibit a larger differentiated contingent (Gleason 3+4)^34^.

We then constructed a second fully annotated TMA from prostatectomy pieces of 101 patients that underwent surgery between 1 February 2010 and 1 December 2011 in the Department of Urology of the University Hospital of Toulouse (Supplementary Tables 3 and 4). The clinical and biochemical (post-operative serum Prostate-Specific Antigen (PSA)) outcomes of the patients were updated in March 2015. CCR3 staining was then performed in this second TMA and scored by two pathologists into low-, intermediate- and high-expressing tumours. Stained areas of all tumours were digitalized and protein expression was quantified using ImageJ software plugins to obtain more accurate measures than the traditional evaluation relying on manual scoring^35^,^36^. We verified that the results obtained were in accordance with the three groups defined by the manual scoring (see Methods section). As shown in Table 1 and Fig. 4b, CCR3 expression was positively correlated with Gleason score, the size of the undifferentiated component (percentage of tumour classified as Gleason 4 or 5), localization at the peripheral zone of the prostate gland, local extension of the prostate cancer (shown by pT stage), the presence of lymphatic emboli and biochemical recurrence (BCR). Of note, CCR3 was significantly over-expressed in tumours from obese patients (body mass index (BMI) 30–35 kg m^−2^, 7.9 % of the cohort). This result probably indirectly indicates that their surrounding PPAT express higher levels of CCL7 since it has been demonstrated that chemokines are able to regulate the expression of their own receptors^37^. We sought to enlarge these results linking CCR3 to prognosis by investigating its role in surgical treatment failure defined either by BCR, locoregional recurrence or distant metastases or by the use of adjuvant radiation or hormonal deprivation therapy, criteria that we used in a previous study^38^. CCR3 values were strongly correlated with surgical treatment failure (Supplementary Fig. 12a). Very interestingly, when compared in patients exhibiting or not surgical treatment failure at 1 year of follow-up within the four Gleason score classes (i.e. < 7, 7 (3+4 or 4+3), >7), CCR3 values were systematically higher in patients with surgical treatment failure regardless of Gleason scores (Supplementary Fig. 12b). Moreover, the differences in CCR3 values were greater in case of favourable Gleason score (<7; Supplementary Fig. 12b). Finally, we observed in a small series of eight patients that CCR3 expression was slightly upregulated in tumour cells present at the invasive front compared with cells from the centre of tumours, this effect being more pronounced in overweight and obese patients (Supplementary Fig. 13). Note that some blood vessels (indicated by blue stars) also express CCR3 but that quantification was only performed in tumour glands (indicated by arrows) under the supervision of two pathologists. These compelling results show for the first time that CCR3 is expressed in prostate cancer and that its expression, amplified in obesity, is correlated both with the occurrence of aggressive prostate cancer, BCR and surgical treatment failure.

### CCR3 has a major role in tumour progression in obese mice

**Figure 5:**
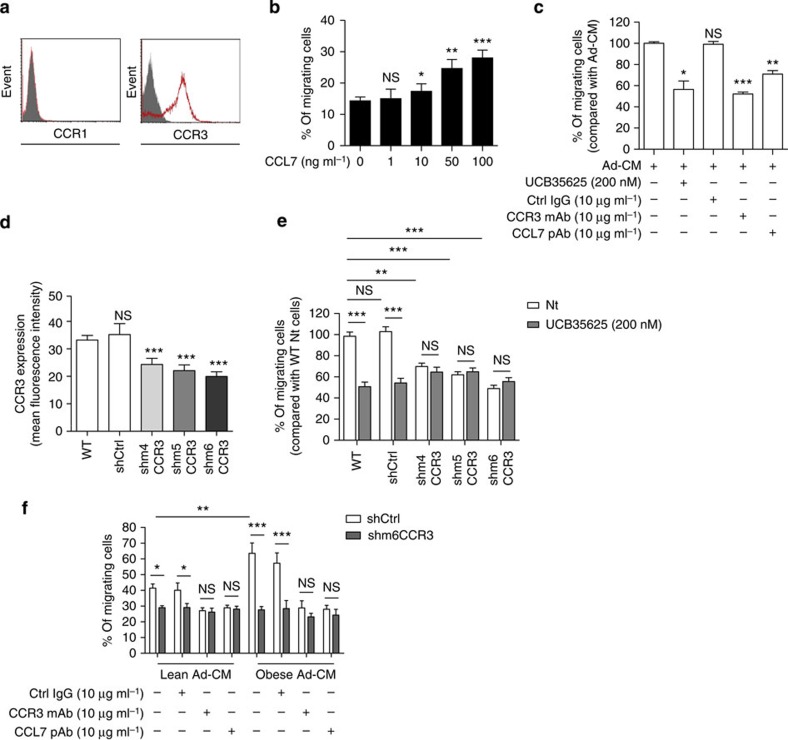
Down-regulation of CCR3 expression in TRAMP-C1P3 cells. (**a**) One representative experiment out of three showing CCR1 and CCR3 expression detected by flow cytometry in the murine prostate cancer cell line TRAMP-C1P3. Solid histogram: control isotypes; open histogram: CCR1 or CCR3. (**b**) *In vitro* migration of TRAMP-C1P3 towards medium without serum, supplemented or not with CCL7 recombinant protein (1–100 ng ml^−1^). Data are shown as mean±s.e.m. (*n*=3). The statistical differences between the mean percentages of migrating cells in experiments performed in the presence *versus* in the absence of CCL7 were evaluated with Student's *t*-tests. (**c**) *In vitro* migration of TRAMP-C1P3 cells towards Ad-CM as a chemoattractant treated or not with UCB35625 (200 nM), anti-CCR3, anti-CCL7 Abs, or control isotype (10 μg ml^−1^). Data are shown as mean±s.e.m. (*n*=3). Data are expressed as the percentage of migrating cells relative to the migration of untreated cells (set to 100%). The statistical significance of differences between means of migrating cells in treated versus untreated cells was evaluated with Student's *t*-tests. (**d**) Mean fluorescence intensity of CCR3 expression determined by flow cytometry in TRAMP-C1P3 cells (WT), transfected with control shRNA (shCtrl) or with one of three different shRNA sequences targeting the CCR3 receptor (shm4, m5 and m6CCR3). Data are shown as mean±s.e.m. (*n*=3). The statistical significance of differences between means of fluorescence in control or CCR3 invalidated cells versus WT cells was evaluated with Student's *t*-tests. (**e**) *In vitro* migration of TRAMP-C1P3 cells transfected with either shCtrl or three different shRNA sequences targeting the CCR3 receptor (shm4, m5 and m6CCR3) towards Ad-CM. As indicated, cells were treated with the CCR3 antagonist UCB35625. Data are shown as mean±s.e.m. (*n*=3). (**f**) I*n vitro* migration of TRAMP-C1P3 cells transfected with either shCtrl or shm6CCR3 towards CM of mu-VAT adipocytes obtained from lean or obese mice. As indicated, cells were treated with Abs against CCR3 or CCL7, or control isotype (10 μg ml^−1^). Data are shown as mean±s.e.m. (*n*=3). Statistical analysis: * statistically significant by Student's *t*-test *P*<0.05, **P<0.01, ***P<0.001, NS, not significant. *n* stands for the number of replicated independent experiments.

We then used an orthotopic graft model in the obesity-sensitive C57BL/6 murine strain^39^,^40^ to assess the importance of the CCR3/CCL7 axis and its regulation by obesity during prostate cancer dissemination *in vivo*. Like all prostate cancer cells studied so far, TRAMP-C1P3 expressed the CCR3 receptor but not CCR1 (Fig. 5a). Recombinant CCL7 protein induced the migration of TRAMP-C1P3 cells and inhibition of the CCR3/CCL7 axis impaired their migration towards Ad-CM (Fig. 5b,c). We engineered TRAMP-C1P3 cell lines stably expressing three different short hairpin RNAs (shRNA) against CCR3 (m4CCR3, m5CCR3 and m6CCR3). CCR3 expression at the cell surface was 27–40% lower in these cells than in wild-type cells (wtCCR3; Fig. 5d). Their migration towards Ad-CM was about twofold lower than that of wtCCR3 cells and migration was the most impaired in the m6CCR3 cell line (Fig. 5e). In contrast with wtCCR3 cells, the migration of shCCR3 cells in response to Ad-CM was not affected by the use of the CCR3 inhibitor, UCB35625 (Fig. 5e). Of note, the migration of shCCR3 cells was similar to wt cells when 10% FCS was used as a chemoattractant, highlighting the specificity towards CCR3 of the observed migration defect (Supplementary Fig. 14). Finally, in contrast with shCtrl cells, the migration of m6CCR3 cells in response to mu-VAT-CM from obese or lean mice was similar and was not affected by the inhibition of the CCR3/CCL7 axis (Fig. 5f), highlighting again the key role of this axis in the increased prostate cancer migration observed in conditions of obesity.

**Figure 6:**
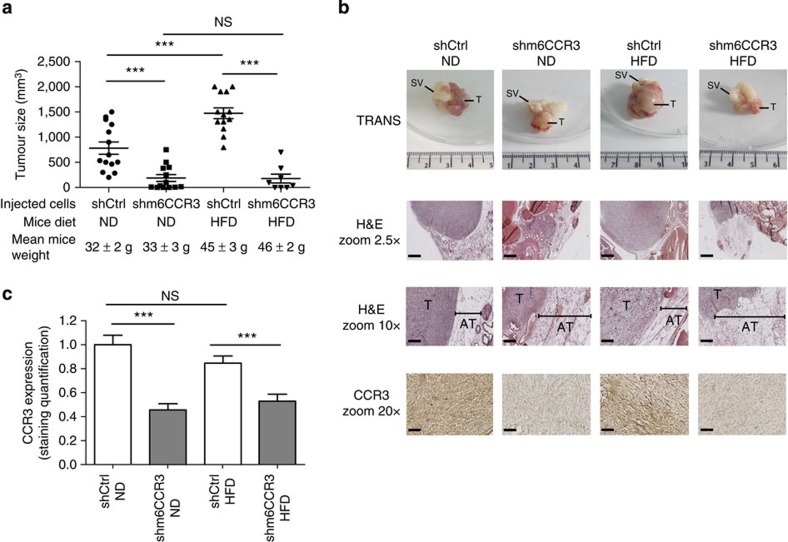
CCR3 contributes to prostate cancer progression *in vivo* and this event is regulated by obesity. (**a**) GFP-tagged shCtrl or m6CCR3 transfected TRAMP-C1P3 cells were injected into the prostate (dorsal lobe) of 18 week-old C57BL/6 mice fed a standard rodent diet (normal diet, ND) or a high-fat diet (HFD). Graphs depict the tumour volume measured 21 days after injection. The mean weight of the mice before the injection, as well as the mean tumour size±s.e.m. in each group, is shown on the legend (*N*⩾8 tumours per group). (**b**) Representative photograph of a tumour from each group taken with white light (Trans) imaging (upper line of the picture; T, tumour; SV, seminal vesicles). The three other lines represent tumour sections from each group after hematoxylin-eosin (H&E) coloration (top and middle, T, tumour; AT, adipose tissue) and CCR3 staining in tumours (lower line). The hypertrophy of adipocytes in obese mice is shown by their comparison with adipocytes from lean mice in mice grafted with the shm6CCR3 cell line (mean size in ND: 56.47±16.63 μm versus mean size in HFD: 133.53±23.82 μm, *P*<0.001, as determined using ImageJ software). Scale bars, respectively, for 2.5, 10 and 20 × : 1 mm, 250 and 125 μm. (**c**) Quantitative expression of CCR3 staining with ImageJ. Data are shown as mean±s.e.m. (three tumours in each group with five fields per tumour). Statistical analysis: *** statistically significant by Student's *t*-test *P*<0.001, NS, not significant.

ShCtrl and m6CCR3 cells were injected into the prostate of C57BL/6 mice. After 3 weeks, tumours formed in animals injected with shCtrl cells were significantly larger in mice fed a high-fat diet (HFD) than in those fed a normal diet (Fig. 6a,b). In both lean and obese conditions, tumours formed in mice injected with cells deficient in m6CCR3 were significantly smaller than in those injected with shCtrl cells (Fig. 6a,b). Furthermore, depletion of CCR3 totally abrogated the differences in tumour size observed between lean and obese mice after injection of the parental cell line. Persistent *in vivo* CCR3 depletion was confirmed by immunohistochemistry on excised tumours (Fig. 6b). We and others have previously demonstrated that when tumour cells come into contact of adipose tissue, adipocytes disappear, fibroblast-like cells accumulate and a desmoplastic stroma ensues, indicating that a cross-talk promoting the tumour's proliferative and invasive capacities is being established^41^,^42^,^43^. In control tumours, PPAT thickness was smaller in areas in contact with tumour and this tissue was almost completely absent in obese animals (Fig. 6b). The observed differences in tumour size for shCtrl cells between obese and lean mice (Fig. 6a) suggest that the cross-talk between adipocytes and tumour cells is amplified in obesity, leading to increased tumour progression. CCR3-deficient tumours were smaller in size, and the surrounding PPAT was still present and thicker than in control tumours, demonstrating that, in these conditions, adipose tissue is not modified by the tumour. At proximity to CCR3-deficient tumours in obese animals, as expected, adipocytes were hypertrophic compared with those of lean mice, increasing PPAT thickness (Fig. 6b, middle panel). Although we assessed that the short-term (up to 5 days) growth of CCR3-deficient cells was similar to wt cells (Supplementary Fig. 15), we cannot formally exclude that long-term CCR3 depletion intrinsically affects tumour growth and survival. In conclusion, in mouse models, our compelling results show that CCR3 is therefore a major determinant of prostate cancer progression by influencing tumour size and adipose tissue remodelling and that its effect is substantially influenced by obesity.

## Discussion

**Figure 7:**
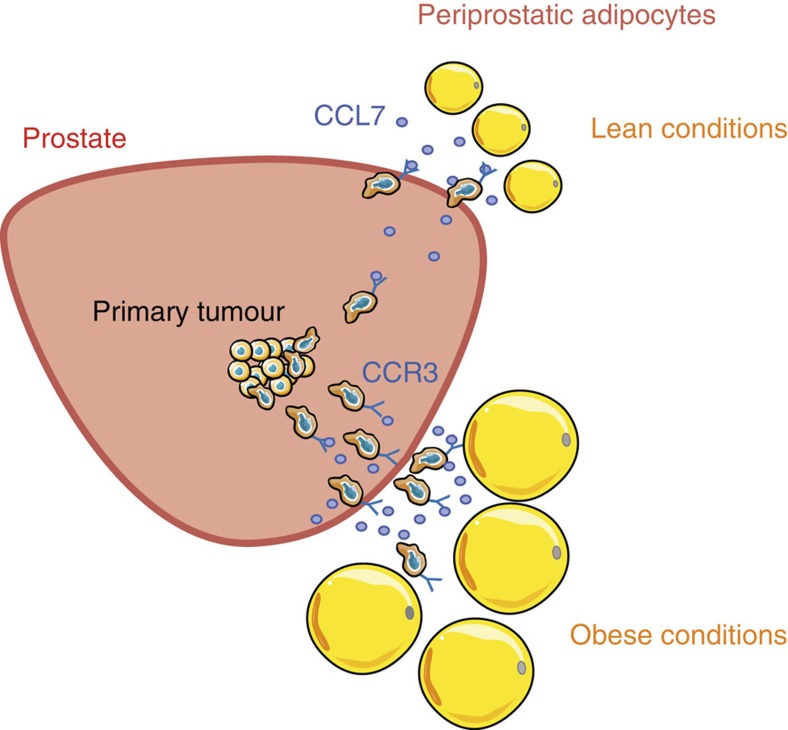
Schematic representation of the proposed role of PPAT in the local dissemination of prostate cancer and its amplification in obesity. Mature adipocytes secrete the chemokine CCL7, which diffuses through the capsule to the peripheral zone of the prostate. The interaction between CCL7 and the CCR3 receptor, expressed by invasive tumour cells, promote their migration outside of the prostate gland. Hypertrophic adipocytes secrete larger amounts of CCL7, which increases adipocyte-dependent directed migration facilitating extraprostatic extension in obesity.

There is increasing evidence in the literature that tumour-surrounding adipocytes might affect tumour progression. When tumour cells invade the adipose tissue, we and others have demonstrated that these invasive cells dramatically impact adipocytes that then exhibit a modified phenotype and specific biological features. We named these adipocytes ‘Cancer-Associated Adipocytes' (CAA)^43^,^44^. In turn, CAAs modify cancer cells' characteristics/phenotype leading to more aggressive behaviour characterized by increased proliferative and invasive capacities, mediated by the secretion of proteases, pro-inflammatory cytokines and modulation of cancer cell metabolism^4^,^22^,^45^. Acquisition of a CAA phenotype has been observed in various cancer types, including prostate, and appears to be a general phenomenon^22^. *In vivo*, morphological changes and delipidation of adipocytes at proximity to tumours are also observed. When adipose tissue is in contact with tumours, this tissue disappears and is replaced by a desmoplastic reaction^42^,^44^,^46^ (see also Fig. 6b). Reaching the surrounding adipose tissue is therefore a key step in tumour progression. At a clinical level, this switch from a prostate-confined tumour to a locally disseminated cancer is also viewed as a crucial step in the progression of the disease^2^. Based on our *in vitro* experiments, we propose that this key step could be controlled by mature adipocytes, which promote the migration of tumour cells to PPAT through chemokine secretion. Ability of tumour cells to migrate towards a chemokine gradient indicates that they have developed a metastatic phenotype (with both migratory and invasive capacities)^8^. Interestingly, acquisition of this phenotype might be also facilitated by surrounding PPAT that expresses inflammatory cytokines, including interleukin-6 (ref. 47), and metalloproteinases^48^, both favoring the invasive properties of tumour cells^49^,^50^,^51^. We used *in vitro* and *in vivo* approaches (murine models) but also human tumours to show that mature adipocytes secrete CCL7, which diffuses through the capsule to the peripheral zone of the prostate. Here CCL7 interacts with the CCR3 receptor to promote cell migration along the chemokine gradient, across the prostate capsule towards PPAT. This axis, with the exception of one *in vitro* study, has not been previously implicated in cancer^52^. Hypertrophic adipocytes secrete large amounts of CCL7, which stimulates adipocyte-dependent directed migration, and then, would facilitate extraprostatic extension in obesity (recapitulated in Fig. 7). The observed increase in migration associated with obesity is totally abrogated when the CCR3/CCL7 axis is inhibited, highlighting its key role in this altered condition. Although the effect of the CCR3/CCL7 axis appears predominant in the enhanced migration of prostate cancer cells, our work underlines that other chemokines and their associated receptors, might be involved in prostate cancer directed migration towards adipose tissue. First, we demonstrated that CXCR2 and CXCR4 also contribute to this effect (Fig. 1c,d), and inhibition of the CXCR4/CXCL12 axis exhibited a slight but significant effect in obese conditions (Supplementary Fig. 11b). Second, in the presence of CXCR2, CXCR4 and CCR3 blocking mAbs, 40% of cells still migrate, highlighting the involvement of other receptors that remain to be identified (Fig. 1d). As reviewed recently^53^, multiple chemokines and their receptors (including, in addition to the receptors tested in our study, CCR5, CCR7, CCR9, CXCR3, CXCR5 and CXCR7) are involved in prostate cancer directed migration. These additional receptors could therefore deserve further investigation in our model. Although not investigated in a context of obesity, a previous study demonstrated that the homing of ovarian cancer cells to the omentum (a large fat pad that extends from the stomach and covers the bowel and the most frequent intra-peritoneal localization of metastatic ovarian cancer) might predominantly involve a CXCR1/CXCL8 axis, at least *in vitro* and in murine models^46^. These results highlight that the homing of tumour cells to surrounding adipose tissue would depend considerably on tumour- and host-dictated characteristics (thar is, the specific secretion pattern of adipose depots). Such a concept is supported by our results with other types of cancer demonstrating, at least *in vitro*, that their migration towards Ad-CM is rather dependent on the CXCR2 and CXCR4 receptors, with no involvement of the CCR3 receptor (Supplementary Fig. 5). The absence of CCR3 involvement in migration towards Ad-CM, despite its expression in other cellular models, is intriguing and is not explained at the present time. It is interesting to note that in contrast to prostate cancer (Supplementary Fig. 7a), the other models have been previously reported secrete CCL7 (refs 29, 54). This autocrine secretion could lead to the absence of a CCL7 gradient between tumour cells and Ad-CM, therefore, hindering directed migration. In addition, it has been previously documented that CCR3 activity exhibits a marked sensitivity to relatively small changes in the extracellular environment (such as pH and ionic strength)^55^ that can potentially be achieved by tumour cell metabolism. This could also represent an interesting hypothesis since it has been proposed that prostate cancer cells might exhibit unique metabolic features with a higher dependence on lipid metabolism than aerobic glycolysis for growth and survival^56^.

In conclusion, deciphering the pivotal chemokine/chemokine receptor axis for adipose tissue-induced chemotaxis in each tumour type would considerably help to set up specific therapeutic strategies especially in obese patients since increased local dissemination is also observed for other cancer types, breast cancer providing the most convincing evidence to date^57^. In human prostate cancer, we showed that the expression of the CCR3 receptor, which is upregulated in obese patients, is associated with aggressive prostate cancer with extended localization and a higher risk of both BCR and treatment failure. To our knowledge, the correlation between expression of a chemokine receptor and these different parameters of prostate cancer progression has never been reported within the same study. Our results highlight the clinical relevance of CCR3 expression in prostate cancer local dissemination. Indeed, first, CCR3 expression is correlated with Gleason score that reflects both the extent of glandular differentiation and the pattern of tumour growth in the prostatic stroma^58^. In fact, higher Gleason scores are characterized by isolated islets of cancer cells that are able to locally disseminate^58^. Second, prostate capsule penetration and local spread, which correspond with pT stage, are also strongly correlated with CCR3 expression. Third, our experiments also suggest that cells expressing higher levels of CCR3 are recruited to the invasive front and this effect is amplified in overweight and obese patients (Supplementary Fig. 13). Therefore, our results suggest new strategies for the treatment of advanced prostate cancer involving CCR3 antagonists, which are currently being developed for other diseases including asthma^17^.

## Methods

### Antibodies

mAbs against CCR1 (clone 141-2, reference D063-3) and CCR3 (clone 444-11, reference D085-3) obtained from MBL International (Woburn, MA, USA) were used for flow cytometry (20 μg ml^−1^) and as blocking antibodies (10 μg ml^−1^) in the migration assays. Anti-CCR2 (clone E68, reference ab32144), used for flow cytometry (20 μg ml^−1^), and anti-CCR3 (clone Y31, reference ab32512), used for immunohistochemistry (15 μg ml^−1^), mAbs were purchased from Abcam (Cambridge, MA, USA). Monoclonal Abs against CXCR1 (Clone 42705, reference MAB330), CXCR2 (clone 48311, reference MAB331) and CXCR4 (clone 44716, reference MAB172) were used for flow cytometry (20 μg ml^−1^) and as blocking antibodies in the migration assays (10 μg ml^−1^), and were obtained from R&D Systems (Minneapolis, MN, USA). mAbs against human CCL7 (Clone 36320, reference MAB282) and pAbs against murine CCL7 (reference AF-456) were used as blocking antibodies in the migration assays (10 μg ml^−1^) were obtained from R&D Systems (Minneapolis, MN, USA). CXCL12 blocking mAbs (Clone 79014) directed against murine CXCL12 were obtained from R&D Systems (Minneapolis, MN, USA) and used for migration assay (10 μg ml^−1^).

### Inhibitors and recombinant chemokines

Recombinant CCL7 protein was obtained from Peprotech (Rocky hill, CT, USA). The antagonist of CXCR1 and CXCR2, SB225002 (ref. 19) was purchased from Tocris (Bristol, UK) and used at final concentrations from 1 to 100 nM. SB225002 inhibits neutrophil chemotaxis in response to CXCL1 and IL8 *in vitro* (IC_50_=20 nM)^19^. The CXCR4 antagonist, AMD3100, which inhibits CXCL12-stimulated chemotaxis (IC_50_=50 nM)^59^, was purchased from Sigma-Aldrich and used at final concentrations from 1 to 100 nM. The inhibitor targeting CCR2, sc-202525, which inhibits CCL2-induced chemotaxis (IC_50_=10 nM)^20^ was purchased from Santa Cruz Biotechnology (Dallas, TE, USA) and used at final concentrations from 1 to 25 nM. The inhibitor targeting CCR1 and CCR3, UCB35625 was purchased from Tocris (Bristol, UK) and used at final concentrations from 1 to 200 nM. This molecule inhibits CCL11-induced chemotaxis in cells transfected with CCR3 (with an IC_50_ value of 93.8 nM)^21^. The maximal doses (between two- and 2.5-fold the IC_50_) do not exhibit toxicity on prostate cancer cells as determined by 3-(4,5-diMethylThiazol-2-yl)-2,5-diphenylTetrazolium bromide (MTT) assays after 24 h exposure (<15% decrease in the number of treated cells compared with untreated control). All the molecules were dissolved in dimethylsulfoxide (DMSO). The final DMSO concentration of each dilution was used in all corresponding controls.

### Cell lines and culture

The human prostate tumour cell lines LNCaP (ATCCCRL-1740), C4-2B (from DSMZ, Braunschweig, Germany), Du-145 (ATCCHTB-81) and PC-3 (ATCCCRL-1435; provided by Dr Olivier Cuvillier, IPBS, Toulouse, France) were used in this study. LNCaP, Du-145 and PC-3 are derived from human prostate carcinoma metastases (lymphonodal, brain and bone lesions, respectively^60^,^61^,^62^). C4-2B cell line was derived from a bone metastasis after orthotopic transplantation of C4-2 cells (subclone of LNCaP) in nude mice^63^.

The human colon carcinoma cell lines sw480 (ATCCCCL-228) and sw620 (ATCCCCL-227; provided by Dr A. Ferrand, CRCT UMR 1037, Toulouse, France), the pancreatic cancer cell line CAPAN (ATCC HTB-79) and PANC- I (ATCCCRL-2547) (provided by Dr C. Bousquet, CRCT UMR1037, Toulouse, France), the breast cancer cell lines T-47D (ATCC HTB-133) and MDA-MB321 (ATCCCRM-HTB-26; provided by Dr K. Bistricky, LBME), the melanoma cell lines 501mel and Lu1205 (gifts from Dr L. Larrue, Institut Curie, Orsay, France ). All cell lines were cultured in RPMI medium (Invitrogen, Auckland, NZ) supplemented with 10% FCS, 125 mg ml^−1^ streptomycin and 125 UI ml^−1^ penicillin, in a humidified atmosphere of 5% CO_2_.

The murine prostate cancer cell line TRAMP-C1P3 (ATCCCRL-2730)^39^ was kindly provided by Dr Richard P. Ciavarra (Eastern Virginia Medical School, Norfolk, VA) and cultured in DMEM medium (Invitrogen, Auckland, NZ) supplemented with 10% FCS, 5 mg ml^−1^ insulin (Sigma-Aldrich, St Louis, MO, USA), 10 nM dihydrotestosterone (Sigma-Aldrich) and antibiotics. The TRAMP-C1P3 was obtained using an *in vivo* selection scheme of intraprostatic implantation of TRAMP-C1 cells, derived from a prostate adenocarcinoma in transgenic C57BL/6 mice expressing the antigen SV40 under the control of the prostate specific probasin promoter^39^. All the cell lines were used within 2 months after resuscitation of frozen aliquots. To obtain TRAMP-C1P3 cells with stable downregulation of CCR3 expression, cells were transduced with the lentiviral vector PLVTMH containing either control or shRNA coding sequences for murine CCR3 (NM_009914). Note that cells transduced with lentiviral vectors also expressed GFP. Interference sequences were generated with the ‘siDesign' Dharmacon tool. The sequences of the sense strands of the shRNAs generated are:

m4CCR3 5′- cgcgtccccAGACCACACCCTATGAATAttcaagagaTATTCATAGGGTGTGGTCTtttttggaaat -3′, m5CCR3 5′- cgcgtccccGACCACACCCTATGAATATttcaagagaATATTCATAGGGTGTGGTCtttttggaaat -3′ m6CCR3 5′- cgcgtccccGGTGAGAGGTTCCGGAAACttcaagagaGTTTCCGGAACCTCTCACCtttttggaaat -3′ under the control of the H1 promoter. Nucleotide sequences targeting CCR3 are shown in capital letters, whereas the sequence responsible for the hairpin structure and sequences necessary for the directional cloning are shown in lowercase letters. 293T cells were kindly provided by Genethon (France) and cultured in Dulbecco's modified Eagle's medium (DMEM) supplemented with 10% FCS. Generation of 293T-LVTHM-shCCR3 and preparation of high-titer lentiviral vector pseudotyped with VSV-G protein was performed as followed. Subconfluent 293T cells were cotransfected with 20 μg of a plasmid vector, 15 μg of pCMV-ΔR8.91, and 5 μg of pMD2G-VSVG by calcium phosphate precipitation. After 16 h medium was changed, and recombinant lentivirus vectors were harvested 24 h later. Cells (50 × 10^3^) were plated on 35-mm dishes 24 h before transduction with viral vectors at a multiplicity of infection of 10:1 (refs 64, 65). One week after transduction and amplification, sterile fluorescence-activated cell sorting was used to select a population consisting of >90% GFP-expressing cells. The cells were kept in culture for a maximum of 1 month and the expression of GFP was checked every week.

The murine 3T3-F442A pre-adipocyte cell line was cultured in DMEM (Invitrogen, Auckland, NZ) supplemented with 10% FCS, 2 mM glutamine and antibiotics. Differentiation was induced by incubating confluent cells in differentiation medium (DMEM supplemented with 10% FCS and50 nM insulin) for up to 14 days after which more than 90% of the cells had accumulated fat droplets.

Ad-CM was obtained by incubating the murine adipocytes in DMEM medium containing no FCS and 1% BSA (Sigma-Aldrich) and, after 12 h, the medium was harvested and immediately stored at −80 °C. Media from a six-well plate in which the cells were differentiated was pooled to create one sample of Ad-CM. The frozen aliquots were only used once, as for all the conditioned media used in our study. For migration assays, the conditioned medium was diluted to one half in DMEM medium containing 1% BSA. Cell lines were authenticated on the basis of viability, recovery, growth rate and morphology. Cell lines were regularly tested for mycoplasma contamination.

### Mice

Mice were handled in accordance with National Institute of Medical Research (INSERM) principles and guidelines. C57Bl6/J male mice were obtained from Janvier (Le Genest St Isle, France). Mice were housed conventionally in an animal room at constant temperature (20–22 °C) and humidity (50–60%), and with a 12 h light–dark cycle. All the mice had free access to food and water throughout the experiment. The C57Bl6/J mice were assigned to a normal diet (PicoLab Rodent Diet 20, Purina Mills, Inc., Brentwood, MO, USA) or HFD (Research Diets, Inc., New Brunswick, New Jersey, USA). The energy contents of the diets were as follows: 16% protein, 81% carbohydrate and 4% fat for the normal diet; 20% protein, 20% carbohydrate and 60% fat for the HFD. C57Bl6/J mice (initially 10 weeks old) were fed a normal diet or a HFD for 8–10 weeks. A 10-week period of HFD is used to mimic the development of obesity in humans associated with the emergence of insulin resistance and low-grade inflammation^66^. To collect visceral adipose tissue (mu-VAT), mice were killed at 20 weeks. For intraprostatic injection of TRAMP-C1P3 cells expressing control (Ctrl) or anti-CCR3 shRNA, we used mice fed either a normal diet or a HFD at 18 weeks. The mice continued to be fed either a normal diet or HFD and were killed 3 weeks later (21 weeks old). The Midi-Pyrénées animal ethics committee approved all procedures.

### Conditioned medium from mu-VAT

Mu-VAT was dissected from lean or obese mice immediately post mortem. Mu-VAT was weighed and 1 g of tissue was placed immediately in 8 ml of DMEM medium supplemented with 1% BSA. Mu-VAT was incubated overnight at 37 °C in a humidified incubator with 5% CO_2_, the medium was collected (mu-VAT-CM) and stored in small aliquots at −80 °C. Mu-VAT-CM was obtained from three animals from each group (lean or obese).

### Conditioned medium from isolated adipocytes and SVF

In particular experiments (see text), mature adipocytes and SVF cells were isolated from whole mu-VAT. Briefly, mu-VAT (1 g of tissue) was incubated in 8 ml of DMEM, 1% BSA in the presence of liberase at a final concentration of 25 μg ml^−1^ (Roche Applied Science, Meylan, France) for 30 min at 37 °C under shaking. After digestion, FCS at a final 5% concentration was added to inhibit the liberase. The samples were then centrifuged (20 min, room temperature, 100 g) to separate adipocytes (floating cells) from the SVF (pellet). Six animals from each group (lean or obese) were used for one experiment. The pooled isolated primary adipocytes and SVF cells were then cultured (5 × 10^6^ cells) in 5 ml of DMEM (without serum) containing 1% BSA for 24 h and the CM was collected at the end of the incubation period and immediately stored at −80 °C.

### Hu-PPAT samples

hu-PPAT samples were collected from radical prostatectomy in accordance with the recommendations of the ethics committee of the Rangueil Hospital (Toulouse, France). All patients gave their informed consent to participate to this study, which was conducted in accordance with the Declaration of Helsinki Principles as revised in 2000. PPAT samples were collected at a distance from the tumours and were macroscopically devoid of fibrosis. These samples were collected and treated within 15 min after the surgery to limit the delay between devascularization and freezing, thus ensuring the preservation of labile molecules. PPAT samples were weighed and 1 g of tissue was placed immediately in 5 ml of DMEM supplemented with 1% BSA. Hu-PPAT was incubated overnight at 37 °C in a humidified incubator with 5% CO_2_ and the medium was collected (hu-PPAT-CM). For each patient (*n*=5), hu-PPAT-CM was separated into small aliquots and immediately stored at −80 °C. In specified experiments, staged biopsies were performed in three independent prostatectomy pieces (punch biopsy with 4 mm trocard). These biopsies were performed in PPAT, at the same level as the prostate capsule and inside the prostate gland (at 2 and 4 cm from the prostate capsule, for biopsy 2 and 3, respectively). After sampling, the tissues were frozen in liquid nitrogen and stored at −80 °C. For CCL7 ELISA assays, tissues were dissociated with a tissue dissociator, which enables the release of secreted substances from the tissue (GentleMACS, Miltenyi Biotec, Inc., Auburn, CA, USA), in 1 ml of buffer containing PBS 1 × and 1% BSA.

### *Ex vivo* differentiation of progenitors cells from the SVF of hu-PPAT

PPAT pieces were submitted to collagenase digestion as previously described in Methods, then centrifuged to separate adipocytes from SVF (pellet). The SVF was then used for *ex vivo* differentiation. Briefly, confluent cells (day 0) were differentiated in DMEM containing 1 μM insulin, 10 μM dexamethasone, 0.5 mM IBMX, supplemented with 10% FCS, 2 mM glutamine and antibiotics. Two days post induction, medium was changed to DMEM containing only 1 μM insulin, 10% FBS with glutamine and antibiotics. After 10–14 days of culture in lipogenic medium, Ad-CM was attained by incubating the obtained mature adipocytes (obtained from human PPAT) in DMEM medium containing no FCS and 1% BSA (Sigma-Aldrich) for 12 h, after which, the medium was harvested, and immediately stored at −80 °C. The media from each six-well plate of differentiated cells was pooled to create one sample of Ad-CM. The frozen aliquots were only used once, as for all the conditioned media used in our study. For migration assays, the conditioned medium was diluted to one half in DMEM medium containing 1% BSA.

### Human visceral adipose tissue

Human visceral adipose tissue (hu-VAT) was collected according to the guidelines of and the full ethical approval from the Ethics Committee of Toulouse Rangueil and Nancy Jeanne d'Arc Hospitals. All patients gave their informed consent to participate in the study and research was conducted in accordance with the Declaration of Helsinki Principles as revised in 2000 (http://www.wma.net/en/30publications/10policies/b3/). Hu-VAT samples from normal weight individuals were obtained from eight patients undergoing intra-abdominal surgery (aged 42.7±4.5 years, BMI 23.1±3.3 kg m^−2^). Hu-VAT from obese individuals was obtained from 18 patients suffering from morbid obesity grade III undergoing bariatric surgery (aged 44.5±1.8 years, BMI 47.6±1.3 kg m^−2^). Tissue samples were immediately frozen in liquid nitrogen after removal and stored at −80 °C. Samples were then used for mRNA extraction and quantitative reverse transcription PCR (RT-qPCR).

### Boyden chamber migration assays

For cell migration, 2 × 10^5^ serum-deprived cells were seeded in the upper chamber of Transwell plates (obtained from Greiner Bio-One, Frickenhausen, Germany, 8 μM pores). The lower chamber was filled with either DMEM without serum, DMEM containing 10% FCS or Ad-CM. Similar experiments were also performed with lower chambers filled with mu-VAT-CM, primary Ad-CM, SVF-CM (from lean or obese mice) or hu-PPAT-CM. After 12 h incubation at 37 °C, non-migrated cells were removed by wiping the upper side of the membranes with a cotton swab. This step was omitted for a control assay to assess the total number of viable cells at the end of the experiment. The transmigrated cells present in the undersurface of the inserts were stained with Toluidine blue 1% supplemented with 0.1 M borax (Sigma, St Louis, MO, USA). Membranes were then cut and incubated in a lysis buffer (Tris-HCl 6.25mm (pH 6.8), 10% glycerol, 2% SDS, 10% β-mercaptoethanol). Absorbance at 570 nm was measured with a 100 μl aliquot in duplicate transferred to a 96-well plate (μQUANT from Biotek Instrument Inc, Winooski, VT, USA). In particular experiments, cells were pre-incubated for 30 min at 37 °C with blocking mAbs directed against CCR3, CCL7 or control IgG at a final concentration of 10 μg ml^−1^ or with pharmacological inhibitors against CXCR1/2, CXCR4, CCR1/CCR3 or CCR2. Experiments with recombinant chemokine CCL7 were also performed. In this case, the chemokine was added to the culture medium containing 0.1% BSA without serum in the lower chamber.

### Flow cytometry and ELISA

For flow cytometry analyses, 1 × 10^6^ prostate tumour cells were fixed using paraformaldehyde solution (3.7% diluted in PBS) during 20 min at 4 °C. After washing with PBS, cells are incubated for 2 h at 4 °C with 10–20 μg ml^−1^ of mAbs (as recommended by the manufacturer) or matched control isotypes at similar concentrations. After washing with PBS containing 0.5% BSA and 2% FCS, cells were incubated for 30 min at 4 °C with secondary fluorescein-labelled IgG. The cells were analysed in a FACScan flow cytometer (Becton Dickinson, Franklin Lanes, NJ). Secreted murine and human CCL7 were quantified with an ELISA kit from Peprotech and R&D Systems, respectively, according to the protocols provided by the manufacturers. Secreted murine CXCL12 was quantified with an ELISA kit from R&D systems, according to the protocols provided by the manufacturers.

### RNA extraction and RTq-PCR

Total RNAs were extracted using the RNeasy mini kit (Qiagen GmbH, Hilden, Germany). Gene expression was analysed using RT-qPCR. Total RNAs (1 μg) were reverse transcribed for 60 min at 37 °C using Superscript II reverse transcriptase (Invitrogen, Auckland, NZ) in the presence of a random hexamer. A minus reverse transcriptase reaction was performed in parallel to ensure the absence of genomic DNA contamination. RT-qPCR was performed starting with 25 ng of cDNA and 300, 500 or 900 nM concentration of a mix of sense/antisense primers (depending on genes) in a final volume of 25 μl using the SYBR Green Universal PCR master mix (Applied Biosystems, Foster City, CA). Fluorescence was monitored and analysed in a GeneAmp 7300 detection system instrument (Applied Biosystems, Foster City, CA). Analysis of housekeeping genes HPRT and GAPDH (500 nM) was performed in parallel to normalize for gene expression. Data were analysed by the 2^*−*ΔΔCt^ method and presented as fold change relative to a control sample after normalization against the expression of housekeeping genes. Here Ct corresponds to the number of cycles needed to generate a fluorescent signal above a predefined threshold. All primers used in this study have been validated for PCR efficiency and are presented in Table 2.

### Analysis of the adipocyte secretome by mass spectrometry

Ad-CM (serum- and phenol red-free) was collected on ice, centrifuged and filtered to remove cell debris and supplemented with complete protease inhibitor cocktail (Sigma-Aldrich). A total of 5 ml of Ad-CM was concentrated with StrataClean resin (Stevens, CA, USA) according to the manufacturer's instructions. After reduction and alkylation of cysteine, the sample was separated by 12% acrylamide SDS–PAGE. Proteins were visualized by Coomassie Blue staining and each lane was cut into 13 homogenous slices and subjected to in-gel tryptic digestion. The tryptic digest was analysed by nano liquid chromatography coupled with tandem-mass spectrometry with an Ultimate3000 system (Dionex, Amsterdam, The Netherlands) coupled to an LTQ-Orbitrap mass spectrometer (Thermo Fisher Scientific, Bremen, Germany)^67^, except that peptides were eluted with a 5–50% gradient of solvent B for 60 min at a 300 nl min^−1^ flow rate. Mascot was used to automatically extract peak lists from raw files. MS/MS data were searched against all entries in the *Mus musculus* database and identified peptides were validated with in-house software^67^, except that 1% false discovery rate was used for validation.

### Surgical orthotopic implantation of TRAMP-C1P3 cells

Intraprostatic grafts of either TRAMP-C1P3 shCtrl or shm6CCCR3 were established in 18 week-old C57BL/6 mice fed either a normal diet or HFD by surgical orthotopic implantation. Mice were anaesthetized by isoflurane inhalation and placed in the supine position. A lower midline abdominal incision was made and 30 μl of tumour cell suspension (2 × 10^6^ cells) was injected into the dorsal lobe of the prostate with a 30-gauge needle and glass syringe. The surgical wound was closed in two layers with 4–0 Dexon interrupted sutures. All procedures were performed with a dissecting microscope. The local Institutional Animal Care and Use Committee approved the experimental protocols described in the study.

### Autopsy and histology

All mice were killed 21 days later. A midline incision was made to access to the abdominal cavity. The tumours were removed *en bloc* with the seminal vesicles and the surrounding adipose tissue, weighed, and a picture of the tumours was taken. Tumours were then fixed in 4% paraformaldehyde and embedded in paraffin. Sections were stained with hematoxylin and eosin to confirm the nature of the disease and to assess extraprostatic extension and the aspect of adipose tissue. CCR3 expression was determined with the protocol described for human samples (see below).

### Patients and prostate cancer TMA

The first TMA, previously developed by the Pathology Department of Toulouse Rangueil Hospital, containing specimens of prostate cancer (*n*=91 in duplicates) and normal epithelium (*n*=10 in duplicates) was used to report CCR3 expression in relation to cancer differentiation^68^. The sum of tumour Gleason scores was recorded for each microarray. The second fully annotated TMA was engineered from prostatectomy pieces of 101 patients that underwent surgery between 1 February 2010 and 1 December 2011 in the Department of Urology of the University Hospital of Toulouse (directed by Professor P. Rischmann). The clinical and biochemical (PSA measurement) follow-up of the patients was updated in March 2015. Surgical treatment consisted of a robot-assisted, open-retropubic radical prostatectomy. Prostatectomy was associated in some cases with ilio-obturator bilateral lymphadenectomy (standard or extended). All patients included in the study had localized disease without metastasis (as assessed by clinical and radiological examinations) at the time of the surgery. All patients signed a consent form for the use of their tissue samples for scientific purposes before surgical intervention. The surgery specimen was treated within 15 min of its removal to limit the delay between devascularization and freezing, thus ensuring the preservation of labile molecules. Tissue samples were placed in cryovials, frozen in liquid nitrogen and stored at −80 °C. Two pathologists (Dr Catherine Mazerolles and Dr Youri Socrier), who were blind to clinical data, independently selected the areas of interest used for the preparation of TMA. Selected tumour areas had a morphology and histological differentiation similar to the tumour Gleason score of the whole tumour or the worst Gleason score found in the tumour in the case of multifocal tumours. After selecting the sample block, 0.2 mm diameter core samples were included according to a predetermined pattern in a recipient block. Gleason scores, pathological and clinical stage, surgical margins (a positive surgical margin was defined as cancer cells in contact with the inked specimen surface), follow-up time and BCR, as defined by the European Association of Urology guidelines by two PSA readings >0.2 ng ml^−1^ (ref. 69), were available for all patients. Surgical treatment failure was defined by patients exhibiting either BCR, locoregional recurrence or distant metastases or by the use of adjuvant radiation or hormonal deprivation therapy, as previously defined^38^.

### Analysis of the expression of CCR3 by immunohistochemistry

Immunohistochemical experiments were performed to detect the expression of CCR3 in prostate cancer TMA. Immunostaining was performed with the EnVision FLEX Mini Kit, High pH (Dako Autostainer/Autostainer Plus; Dako France, Trappes, France). The TMA were immersed in xylene to remove paraffin and then rehydrated by successive baths of graded alcohol (100–70% and then distilled water), followed by treatment with the antigen unmasking solution for antigen retrieving (Citrate Target Retrieval Solution from Dako) in a water bath at 95 °C. After saturation of endogenous peroxidases (Peroxidase blocking solution from Dako), the samples were incubated with the primary anti-CCR3 antibody (diluted to 1/100). TMAs were then incubated with FLEX/HRP conjugate secondary antibody (EnVision FLEX+ Mouse and Rabbit from Dako). The samples were rinsed, then treated with liquid DAB (BioGenex, San Ramon, CA) and washed with distilled water. Finally, the counter-staining was carried out with hematoxylin (Dako) and the various TMA were mounted with Eukitt reagent. Two pathologists, who were blind to clinical data, independently scored CCR3 expression in human tumours as negative, low, moderate (mid) or high (manual scoring). None of the tumours were negative for CCR3 expression. The slides were then digitally scanned via Hamamatsu Nanozoomer 2.0RS and analysed with the device software provided by the manufacturer. The intensity of CCR3 staining has been quantified by computer through the use of ImageJ software, with tumour staining separated using deconvolution plug-in^35^,^36^. As a first step, we used a color deconvolution technique to separate the pure DAB and hematoxylin stained areas leaving a complimentary image. The pixel intensities of separated DAB or hematoxylin images range from 0 to 255. Value 0 represents the lightest shade of the color while 255 represent the darkest shade of the color in the image. To assign an automated score by judging the pure DAB staining pattern, a histogram profile of every image, that is, the number of pixels of a specific intensity value versus their respective intensity was raised using ImageJ standard programme feature. Note that we excluded the pixel intensity values corresponding to the unspecific staining. Intensity for each tumour gland was quantified after manual selection of the area of interest under the supervision of pathologists. Finally, we verified that the results obtained were in accordance to the three groups defined by the manual scoring (median signal±s.e.m., low expression: 28.5±4.1, moderate: 46.2±2.0, high: 56.0±3.1 arbitrary units).

### Expression of CCR3 in pT3 human tumours

Frozen tissues obtained after radical prostatectomy of eight patients with pT3a or pT3b prostate cancer, defined by two pathologists (Dr Catherine Mazerolles and Dr Youri Socrier), were analysed for CCR3 expression. Four of these patients presented BMI<25 and four BMI>25. All these patients underwent surgery between 1 February 2010 and 1 December 2011 in the Department of Urology of the University Hospital of Toulouse (directed by Professor P. Rischmann) and signed a consent form for the use of their tissue samples for scientific purposes before surgical intervention. The staining of CCR3 and slides digitalization has been performed as previously. Note that some blood vessels also express CCR3 but that quantification was only performed in tumour glands under the supervision of two pathologists.

### Analysis of cell number by thiazolyl blue tetrazolium bromide (MTT) assay

PC-3 or TRAMP-C1P3 cells transfected with either a control vector (shCtrl) or an sh directed against CCR3 (shm6CCR3) were plated (3 × 10^3^ cells) in quadruplicate for each time point in a 96-well plate. Following the attachment of all cells after 6 h, the number of viable cells was measured by an MTT assay. Briefly, 100 μl of MTT (50 mg ml^−1^) was added to the wells and incubated for 2 h. After aspiration, 100 μl of DMSO was added to each well and incubated for 15 min at 37 °C to solubilize the bio-reduced coloured MTT-formazan and to lyse the cells. The optical density was read at 570 nm in a microplate reader.

### Statistical analysis

The statistical significance of differences between means was evaluated with unpaired Student's *t*-tests. All statistical tests were two-sided. *P* values below 0.05 (*), <0.01 (**) and <0.001 (***) were deemed as significant and ‘NS' was used to denote not significant. For the analysis of TMA, we first performed a descriptive analysis with the clinical and biological features of patients. We then examined the clinical and biological characteristics of patients to identify patients with aggressive and extended tumours. For the correlation between CCR3 expression and quantitative data (for example, prostate weight) or variables with more than two ordered classes regarding disease severity (for example, Gleason score or tumour stage), we used a Spearman's rank correlation test assuming a monotonic relation between considered variables. We used the Student's *t*-test to correlate CCR3 expression to non-ordered variables represented by a category (for example, presence or absence of lymphatic emboli). Non-parametric tests are more conservative than parametric tests. In some cases, we also expected a lack of statistical power given the limited size of some sub-populations of variables. Leptin and CCL7 expression in hu-VAT were correlated using Pearson's correlation analysis.

## Additional information

**How to cite this article:** Laurent, V. *et al*. Periprostatic adipocytes act as a driving force for prostate cancer progression in obesity. *Nat. Commun.* 7:10230 doi: 10.1038/ncomms10230 (2016).

## Supplementary Material

Supplementary Information Supplementary Figures 1-15 and Supplementary Tables 1-4

## Figures and Tables

**Table 1:** Histological and immunohistochemical characteristics of the cohort.

	**CCR3 quantifications (arbitrary units), median (extant)**	***P*** **value**
Prostatectomie piece weight (g)	48.5 (11.4–94.7)	0.82
PSA before surgery (ng ml^−1^)	48.5 (11.4–94.7)	0.38

*Gleason score*		
Gleason <7	27.2 (11.4–85.0)	<0.001
Gleason =7 (3+4)	47.0 (21.0–90.2)	
Gleason =7 (4+3)	52.2 (30.6–75.7)	
Gleason >7	57.0 (44.7–94.7)	

*Tumour localization*		
Transition zone	37.6 (11.4–62.8)	0.04*
Peripheral zone	49.7 (17.3–94.7)	
Percentage of low differentiated contingent (grade 4 and 5)	48.5 (11.4–94.7)	<0.001

*pT stage*		
pT2b	39.9 (31.9–71.1)	0.03
pT2c	41.4 (11.4–75.7)	
pT3a	51.6 (17.3–90.2)	
pT3b	60.0 (35.9–94.7)	

*Lymphatic emboli*		
No	47.4 (11.4–90.2)	0.02*
Yes	62.0 (44.7–94.7)	

*Lymph node invasion*		
Nx (no node dissection performed)	43.4 (18.7–71.1)	0.40*
N0	49.6 (11.4–94.7)	
N1	56.4 (44.7–71.1)	

*Biochemical recurrence*		
No	42.1 (11.4–90.2)	<0.001*
Yes	61.5 (42.2–94.7)	

*Body mass index (BMI)*		
Lean (BMI≤30 kg m^−2^)	47.4 (11.4–85.0)	0.01*
Obese (BMI>30 kg m^−2^)	64.6 (27.7–94.7)	

PSA, prostate-specific antigen; TMA, tissue microarray.

CCR3 expression was evaluated by immunohistochemistry on a TMA containing 101 tumours in duplicate. For the correlation between CCR3 expression and quantitative data (for example prostate weight) or variables with more than two ordered classes regarding disease severity (for example Gleason score or tumour stage), we used Spearman correlation tests assuming a monotonic relation between considered variables. We used Student's *t*-tests (notified by *) to correlate CCR3 expression to non-ordered categorical variables (for example presence or not of emboli or biochemical recurrence). The biochemical recurrence, as defined by the European Association of Urology guidelines, corresponded to two PSA readings >0.2 ng ml^−1^.

**Table 2:** Primers used for RT-qPCR analysis.

**Genes**	**Species**	**Forward primer**	**Reverse primer**	**[C]**
*CCL7*	Murine	5′- AAGATCCCCAAGAGGAATCTCAA -3′	5′- CTTCCCAGGGACACCGACTA -3′	900 nM
*CCL7*	Human	5′- AAACCTCCAATTCTCATGTGGAA -3′	5′- CAGAAGTGCTGCAGAGGCTTT -3′	900 nM
*Leptin*	Murine	5′- GGGCTTCACCCCATTCTGA -3′	5′- TGGCTATCTGCAGCACATTTTG -3′	900 nM
*Leptin*	Human	5′- CCTTCCAGAAACGTGATCCAA -3′	5′- GGCCAGCACGTGAAGAAGAT -3′	900 nM
*Adiponectin*	Murine	5′- TGGAATGACAGGAGCTGAAGG -3′	5′- TATAAGCGGCTTCTCCAGGCT -3′	300 nM
*Adiponectin*	Human	5′- GCAGAGATGGCACCCCTG	5′- GGTTTCACCGATGTCTCCCTTA -3′	900 nM
*TNF-α*	Murine	5′- TGGGACAGTGACCTGGACTGT -3′	5′- TTCGGAAAGCCCATTTGAGT -3′	300 nM
*TNF-α*	Human	5′- CCGAGTCTGGGCAGGTCTAC -3′	5′- TGGGAAGGTTGGATGTTCGT -3′	300 nM
*GAPDH*	Murine and Human	5′- TGCACCACCAACTGCTTAGC -3′	5′- GGCATGGACTGTGGTCATGAG -3′	500 nM
*HPRT*	Murine and Human	5′- TGGCCATCTGCCTAGTAAAGC -3′	5′- GGACGCAGCAACTGACATTTC -3′	500 nM

RT-qPCR, quantitative reverse transcription PCR.
