# Supplementary material for: Periprostatic adipocytes act as a driving force for prostate cancer progression in obesity
Source: Nat Commun. 2016 Jan 12;7:10230. doi: 10.1038/ncomms10230 (PMC4729927; doi:10.1038/ncomms10230)
Supplement: Supplementary Information — Supplementary Figures 1-15 and Supplementary Tables 1-4 [file ncomms10230-s1.pdf]

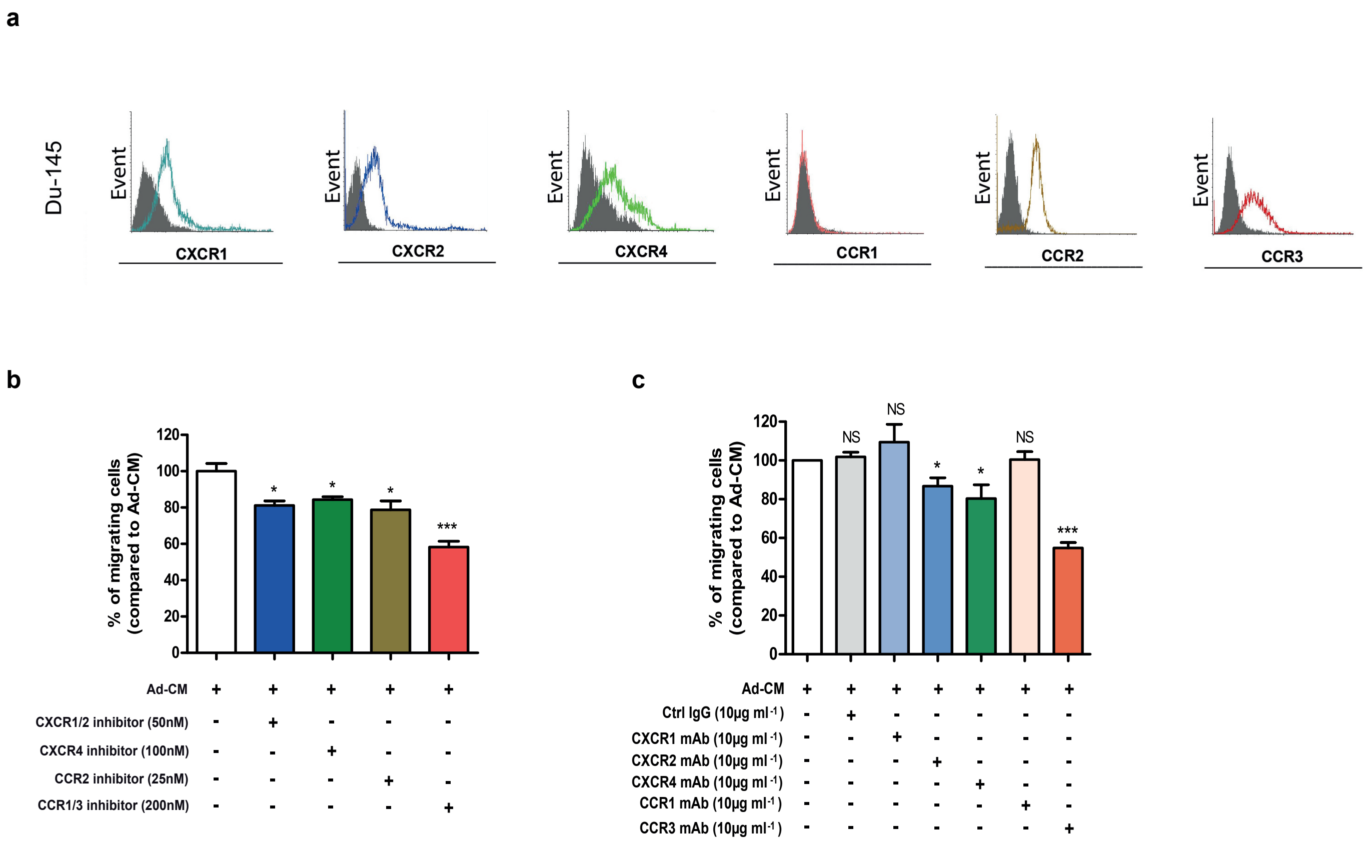

Supplementary Figure 1. CCR3 is involved in the migration of Du-145 cells towards Ad-CM. (a) One representative experiment out of three showing the expression of chemokine receptors (CXCR1, CXCR2, CXCR4, CCR1, CCR2 and CCR3) detected by flow cytometry in the human prostate cancer cells, Du-145. Solid histogram: control isotypes; open histogram: indicated antigens. **(b)** Du-145 cells were pre-incubated or not with the SB225002 (CXCR1/2 inhibitor, 50nM), AMD3100 (CXCR4 inhibitor, 100nM), sc-202525 (CCR2 inhibitor, 25nM) or UCB35625 (CCR1/3 inhibitor, 200nM) for 30 minutes and migration towards Ad-CM (obtained from *in vitro* differentiated 3T3-F442A mature adipocytes) was performed for 12 h in the presence of inhibitors. **(c)** Similar experiments were performed in the presence of cells treated or not with control isotype, anti-CXCR1, anti-CXCR2, anti-CXCR4, anti-CCR1 or anti-CCR3 mAbs, all used at 10µg ml^{-1}. Data are expressed as the percentage of migrating cells relative to the migration of untreated cells (set to 100%) and are shown as mean±s.e.m (n=3). The statistical significance of differences between means of migrating cells (in %) in treated *versus* untreated (NT) cells was evaluated with Student's t-tests. Statistical analysis: * statistically significant by Student's t-test, $p<0.05$, *** $p<0.001$, NS for not significant. n stands for the number of replicated independent experiments.

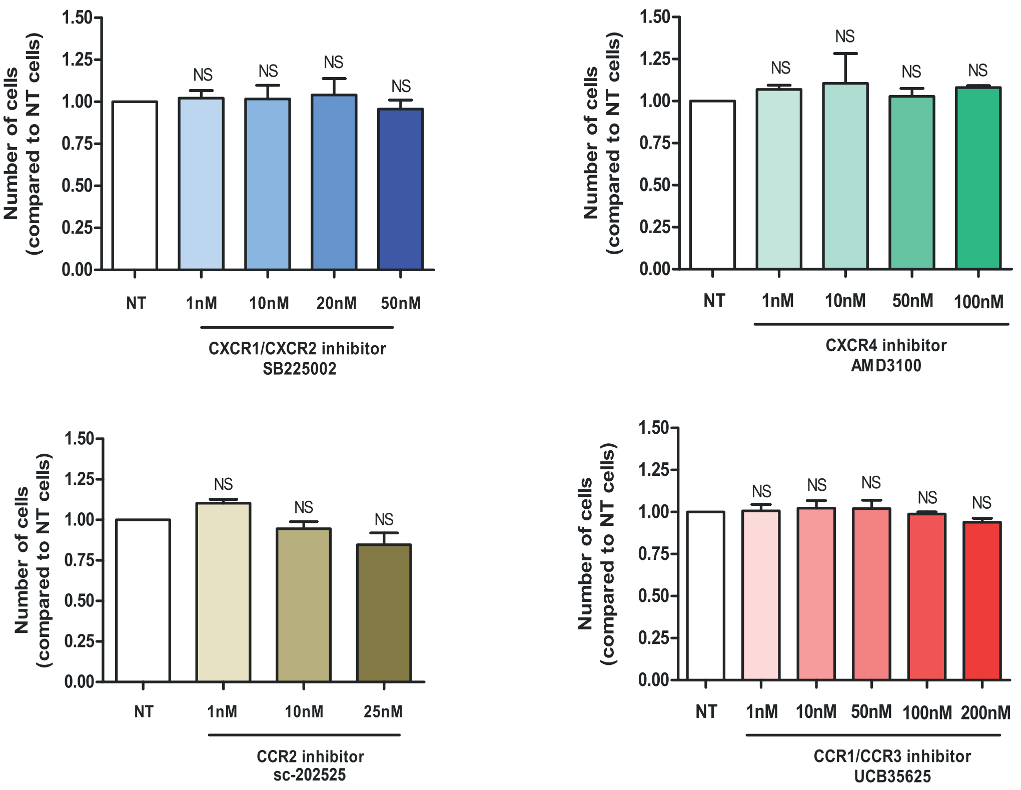

Supplementary Figure 2. Pharmacological inhibitors used in migration assays are not toxic for prostate tumor cells. The effect of the chemokine receptors inhibitors on PC-3 cells viability was determined using MTT assays. Cell viability was determined with cells exposed during 24 h to increasing doses of the pharmacological inhibitors. Data are expressed as the number of treated viable cells relative to the viability of untreated cells (set to 1) and are shown as mean±s.e.m (n=3). The statistical significance of differences between means of viable cells (in %) in treated *versus* untreated (NT) cells was evaluated with Student's t-tests. Statistical analysis: NS for not significant. n stands for the number of replicated independent experiments.

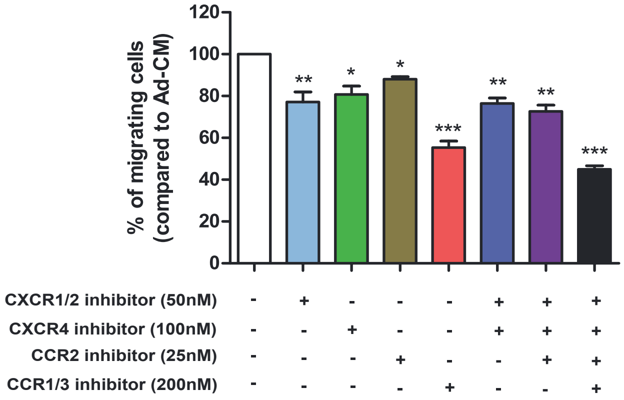

Supplementary Figure 3. CCR3 receptor is a key player in prostate cancer cell migration towards Ad-CM compared to CXCR2, CXCR4 and CCR2. PC-3 cells were pre-incubated or not with the SB225002 (CXCR1/2 inhibitor, 50nM), AMD3100 (CXCR4 inhibitor, 100nM), sc-202525 (CCR2 inhibitor, 25nM) or UCB35625 (CCR1/3 inhibitor, 200nM) for 30 minutes and migration towards Ad-CM (obtained from *in vitro* differentiated 3T3-F442A mature adipocytes) was performed for 12 h in the presence of inhibitors (used alone or in combination). Bar plots represent the percentage of migrating cells relative to the migration of untreated cells (set to 100%). Data are shown as mean±s.e.m (n=3). The statistical significance of differences between means of migrating cells (in %) in treated *versus* untreated (NT) cells was evaluated with Student’s t-tests. Statistical analysis: Statistical analysis: * statistically significant by Student's t-test $p<0.05$, ** $p<0.01$, *** $p<0.001$, NS for not significant. n stands for the number of replicated independent experiments.

a

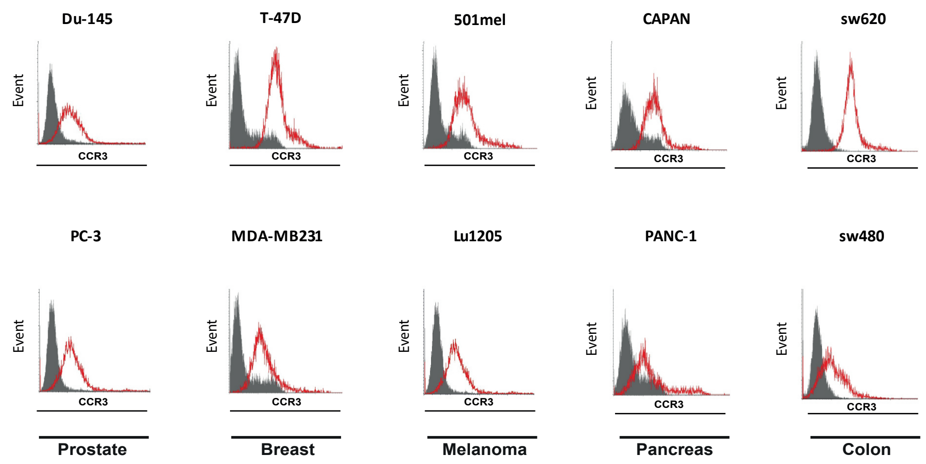

b

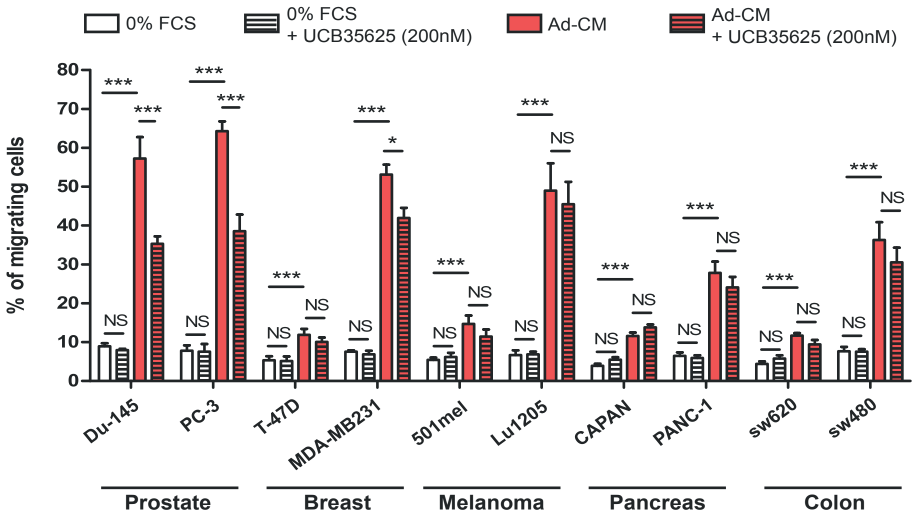

Supplementary Figure 4. CCR3 is not involved in the migration of other cancer models towards Ad-CM. (a) One representative experiment out of three showing CCR3 expression detected by flow cytometry in human prostate cancer (Du-145 and PC-3), breast cancer (T-47D and MDA-MB231), melanoma (501mel and Lu1205), pancreatic cancer (CAPAN and PANC-1) and colon cancer (sw620 and sw480) cell lines. Solid histogram: control isotypes; open histogram: CCR3. CCR1 expression was not detected in all the cell lines (data not shown) **(b)** *In vitro* migration towards either 0% FCS or Ad-CM (*in vitro* differentiated 3T3-F442A mature adipocytes) of human prostate cancer (Du-145 and PC-3), breast cancer (T-47D and MDA-MB231), melanoma (501mel and Lu1205), pancreatic cancer (CAPAN and PANC-1) and colon cancer cells (sw620 and sw480) treated or not with UCB35625 (200nM). Cells were pre-incubated with the inhibitor for 30 minutes and the inhibitor remained present during the migration (12 h for prostate, breast, pancreas, colon cancer models and 24 h for melanoma cells). For each cancer model a poorly aggressive cell line (T-47D, 501mel, CAPAN and sw620) and an aggressive cell line (MDA-MB231, Lu1205, PANC-1 and sw480) were chosen. Note that the migration of aggressive cells towards Ad-CM was higher than poorly aggressive cells. The migration towards 0% FCS that relied on chemokinesis was not affected by CCR3 inhibitor in all tested models. CCR3 inhibitor affects the migration towards Ad-CM of prostate cancer cell lines and of one breast cancer cell line MDA-MB231 (to a slight extent, 20% inhibition). The migration of the other models was unaffected by CCR3 antagonist. Data are shown as mean±s.e.m (n=3). The statistical significance of differences between means of migrating cells (in %) in treated *versus* untreated (NT) cells was evaluated with Student's t-tests. Statistical analysis: * statistically significant by Student's t-test p<0.05, *** p<0.001, NS for not significant. n stands for the number of replicated independent experiments.

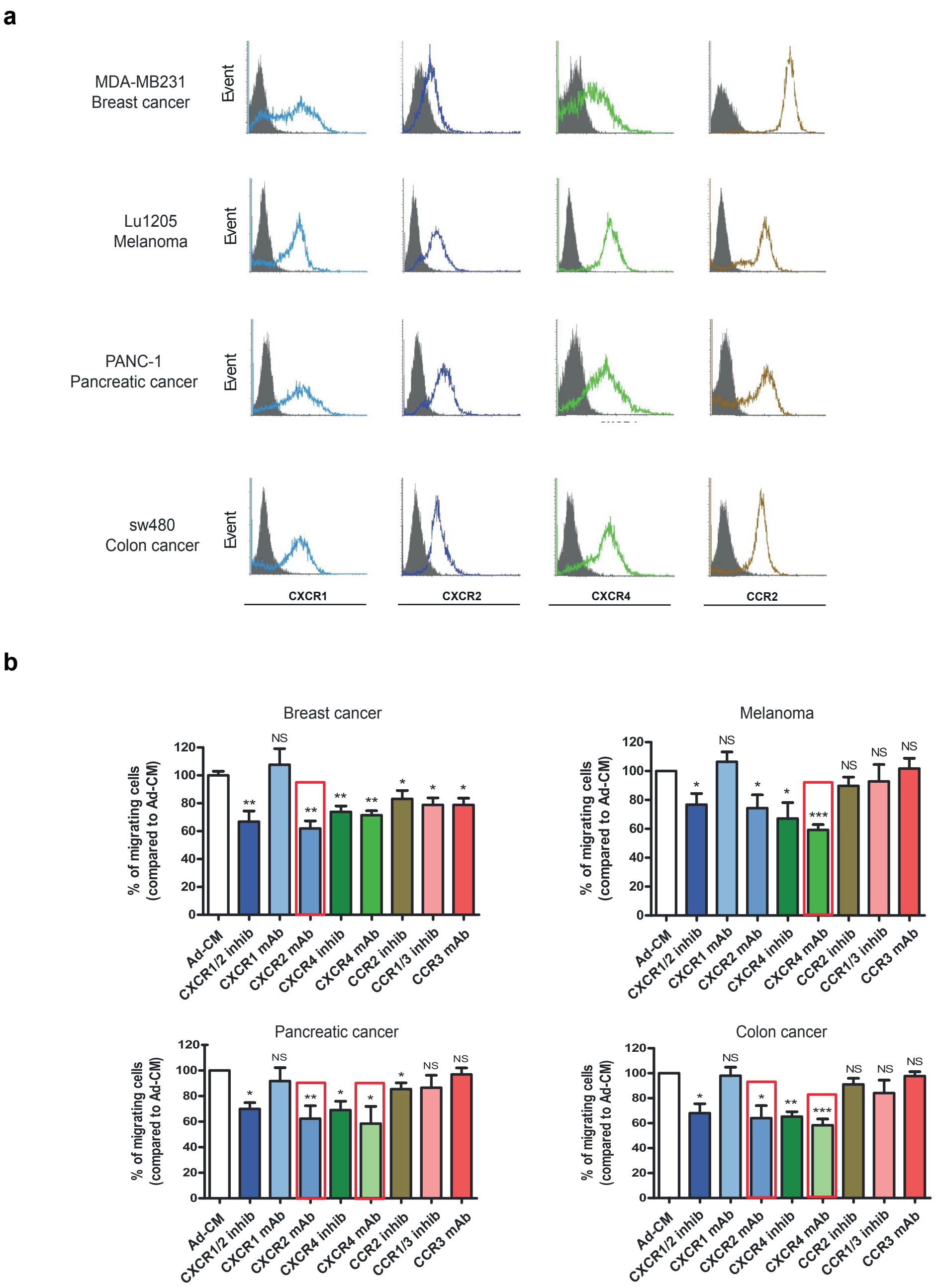

Supplementary Figure 5. Migration of breast, pancreatic, colon cancers and melanoma towards Ad-CM predominantly involves CXCR2 or CXCR4 receptors. (a) One representative experiment out of three showing CXCR1, CXCR2, CXCR4 and CCR2 expression detected by flow cytometry in human breast cancer (MDA-MB231), melanoma (Lu1205), pancreatic cancer (PANC-I) and colon cancer (sw480) cell lines. Solid histogram: control isotypes; open histogram: chemokine receptor expression. **(b)** *In vitro* migration towards Ad-CM (*in vitro* differentiated 3T3-F442A mature adipocytes) of human breast cancer (MDA-MB231), melanoma (Lu1205), pancreatic cancer (PANC-I) and colon cancer cells (sw480) treated or not with the SB225002 (CXCR1/2 inhibitor, 50nM), AMD3100 (CXCR4 inhibitor, 100nM), sc-202525 (CCR2 inhibitor, 25nM) or UCB35625 (CCR1/3 inhibitor, 200nM), with control isotype, anti-CCR1, anti-CCR3, anti-CXCR1, anti-CXCR2 or anti-CXCR4 mAbs, all used at 10µg ml^{-1}. Cells were pre-incubated with the inhibitors or mAbs for 30 minutes and the inhibitors or mAbs remained present during the migration experiments (12 h for prostate, breast, pancreas, colon cancer models and 24 h for melanoma cells). The line surrounding the histogram indicates the mAb that induces the highest inhibitory effect in cell migration. Bar plots represent the percentage of migrating cells relative to the migration of untreated cells (set to 100%). Data are shown as mean±s.e.m (n=3). The statistical significance of differences between means of migrating cells (in %) in treated *versus* untreated (NT) cells was evaluated with Student's t-tests. Statistical analysis: * statistically significant by Student's t-test p<0.05, ** p<0.01, *** p<0.001, NS for not significant. n stands for the number of replicated independent experiments.

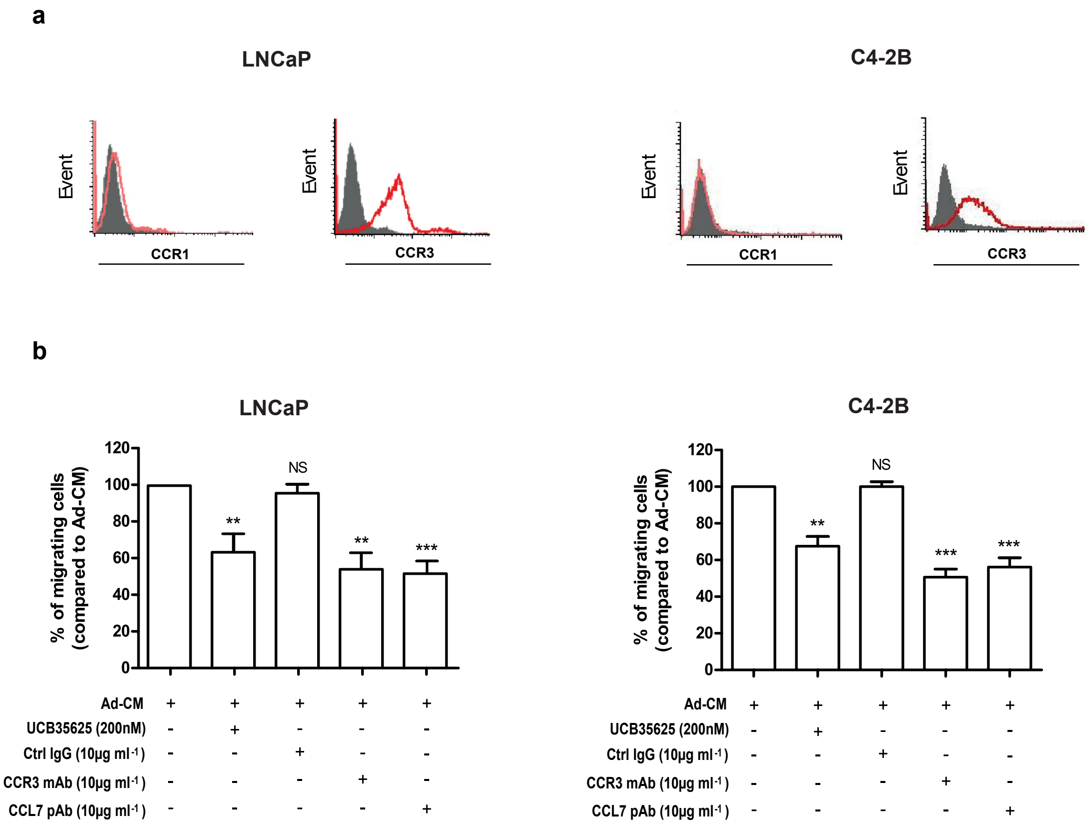

Supplementary Figure 6. CCR3/CCL7 axis is involved in the migration of the poorly aggressive prostate cancer cell lines, LNCaP and C4-2B, towards Ad-CM. (a) One representative experiment out of three showing the expression of chemokine receptors (CCR1 and CCR3) detected by flow cytometry in the human prostate cancer cells, LNCaP and C4-2B. Solid histogram: control isotypes; open histogram: indicated antigens. **(b)** Migration of LNCaP and C4-2B cells towards Ad-CM in the presence or not of UCB35625 (200nM), or control IgG or m/pAbs directed against CCR3 or CCL7 (10µg ml^{-1}). Data are shown as mean±s.e.m (n=3). The statistical significance of differences between means of migrating cells (in %) in treated *versus* untreated (NT) cells was evaluated with Student's t-tests. Statistical analysis: ** statistically significant by Student's t-test p<0.01, *** p<0.001, NS for not significant. n stands for the number of replicated independent experiments.

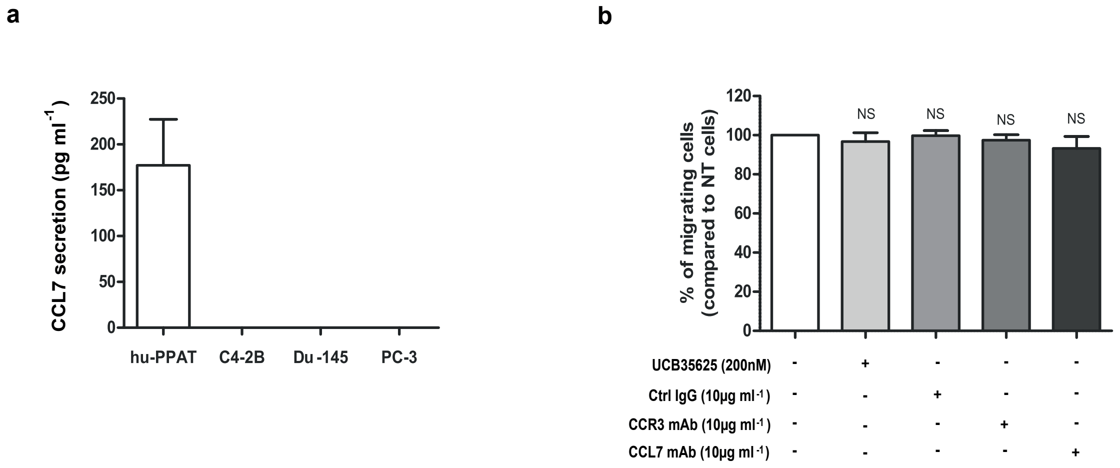

Supplementary Figure 7. Absence of autocrine secretion of CCL7 in prostate cancer cells. (a) Dosage of CCL7 by ELISA in the CM of indicated prostate cancer cell lines. CM was obtained by incubating prostate tumor cells overnight in medium without serum containing 1% BSA. At the end of the incubation period, the medium was collected and stored in small aliquots at -80°C. The secretion of CCL7 is shown as mean±s.e.m (n=3) and hu-PPAT CM is used as a positive control. **(b)** *In vitro* migration of PC-3 cells towards a medium containing 10% FCS used as a chemoattractant treated or not with UCB35625 (200nM), anti-CCR1 or anti-CCR3 mAbs, anti-CCL7 pAbs, or control isotype (all used at 10µg ml^{-1}). Data are shown as mean±s.e.m (n=3). The statistical significance of differences between means of migrating cells (in %) of treated *versus* untreated cells (NT) was evaluated with Student's t-tests. Statistical analysis: NS for not statistically significant. n stands for the number of replicated independent experiments.

a

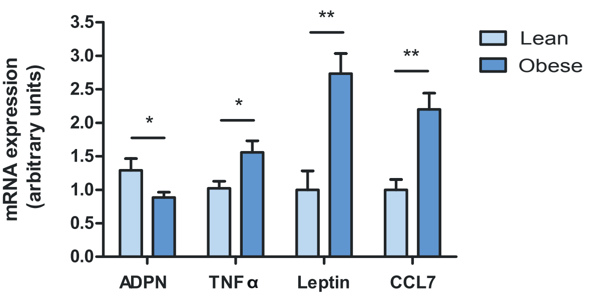

b

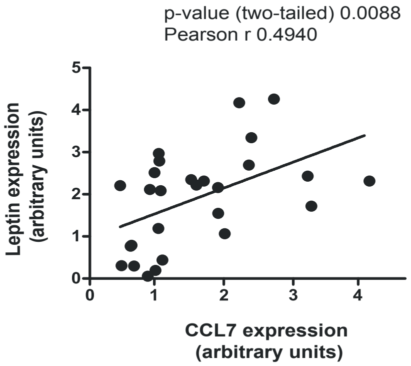

Supplementary Figure 8. CCL7 expression is up-regulated in human VAT in obesity. (a) mRNA were extracted from human visceral adipose tissue (hu-VAT) of either lean (N=8) or obese (N=18) patients undergoing abdominal surgery. Expression of CCL7 mRNA was evaluated by RT-qPCR. A panel of genes whose expression has been shown to decrease (Adiponectin, ADPN) or increase (TNFα, Leptin) in obesity was used as a control to validate the samples. Data are shown as mean±s.e.m. Statistical analysis: * statistically significant by Student's t-test; $p < 0.05$, ** $p < 0.01$. **(b)** Pearson's correlation between leptin (marker of adipocytes hypertrophy) and CCL7 expression. The correlation between leptin and CCL7 expression suggests that CCL7 secretion is related to the hypertrophic state of adipocytes.

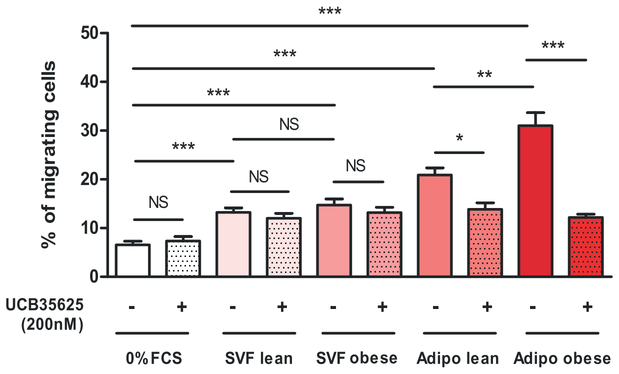

Supplementary Figure 9. The directed migration of prostate cancer cells towards the secretions of SVF does not involve the CCL7/CCR3 axis. *In vitro* migration of PC-3 cells treated or not with the CCR3 inhibitor (UCB35625, 200nM) towards medium without serum (negative control) or CM obtained from adipocytes or SVF cells isolated from the VAT of C57BL/6 lean or obese mice (six animals per group). Data are shown as mean±s.e.m (n=3). Statistical analysis: * statistically significant by Student's t-test $p<0.05$, ** $p<0.01$, *** $p<0.001$, NS for not significant. n stands for the number of replicated independent experiments. Note that the % of migrating cells was significantly higher when SVF-CM was used as a chemoattractant compared to 0% serum. The migration of prostate cancer cells towards SVF-CM was unaffected by obesity and CCR3 antagonist.

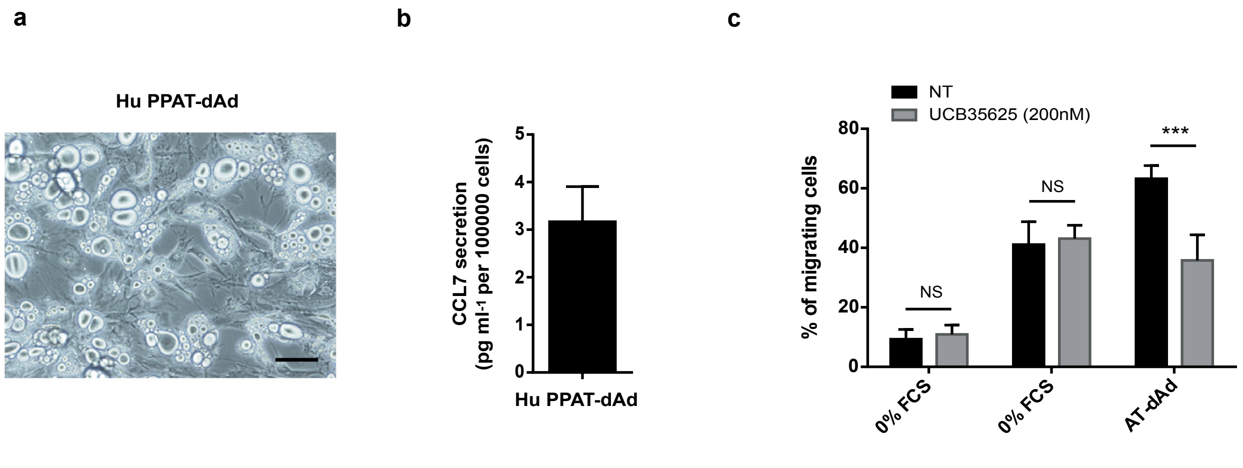

Supplementary Figure 10. *Ex vivo* differentiated mature adipocytes from ADSCs present in the SVF of PPAT secrete CCL7 and are able to support prostate cancer directed migration in a CCR3 dependent manner. (a) Representative image of mature adipocytes obtained after *ex vivo* differentiation of ADSCs isolated from human PPAT (hu-PPAT-dAd). Scale bars, 50µm. **(b)** CCL7 secretion by *ex vivo* differentiated adipocytes. Data are shown as mean±s.e.m (n=3). **(c)** *In vitro* migration towards Ad-CM obtained from *ex vivo* differentiated adipocytes in the presence of UCB35625 (200nM). Cells were pre-incubated with the receptor antagonist for 30 minutes at 37°C prior to their addition to Transwell chambers. The receptor antagonist was also present during the migration assay (12 h). Data are shown as mean±s.e.m (n=3). Statistical analysis: * statistically significant by Student's t-test $p<0.05$, ** $p<0.01$, *** $p<0.001$, NS for not significant. n stands for the number of replicated independent experiments.

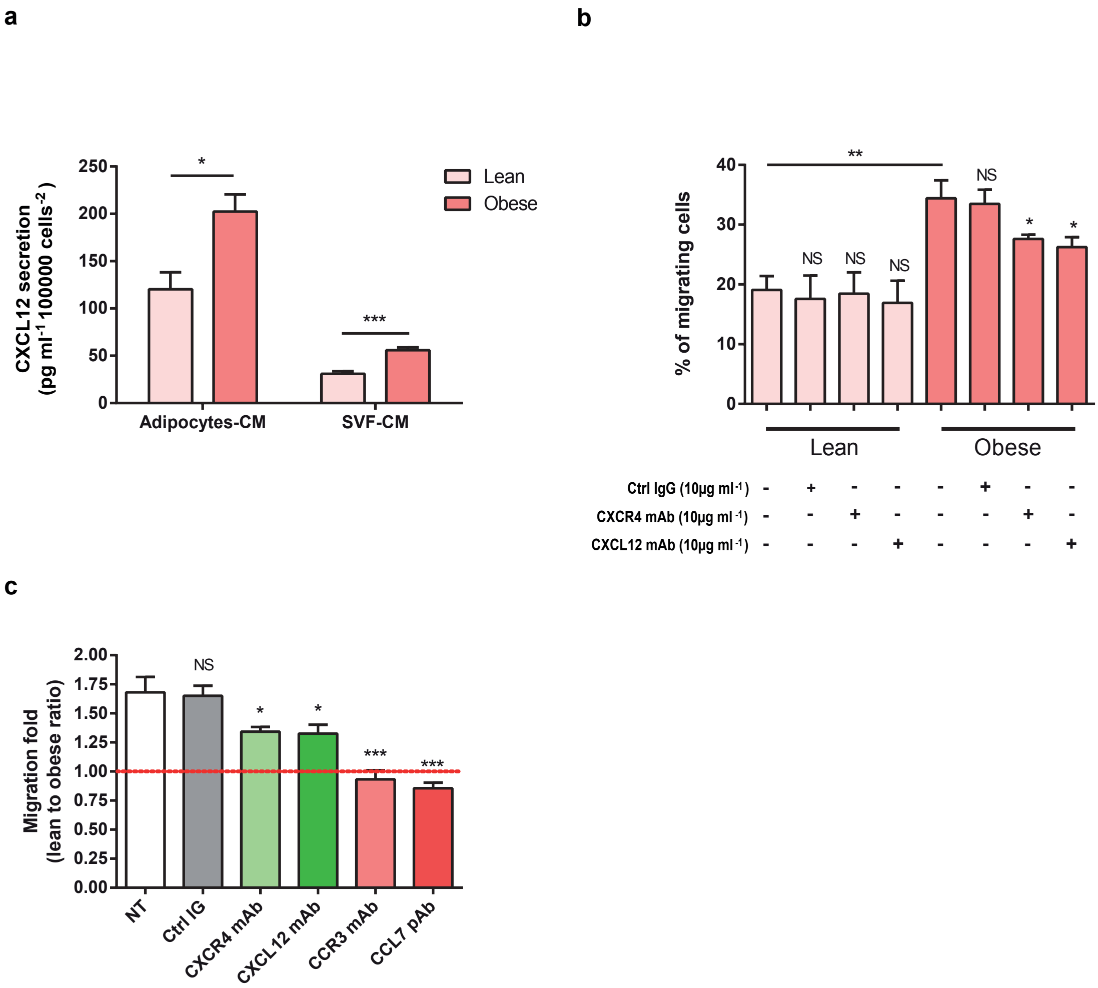

Supplementary Figure 11. CXCR4/CXCL12 axis is poorly involved in the migration towards Ad-CM obtained from lean and obese mice. (a) CXCL12 secretion by primary adipocytes or SVF cells isolated from mu-VAT of C57BL/6 lean or obese mice (six animals per group). Data are shown as mean±s.e.m (n=3). **(b)** Adipocytes were isolated from the mu-vat of lean or obese C57bl6 mice (six animals per group). *In vitro* migration of PC-3 cells towards mu-VAT adipocyte-CM was performed in the presence or absence of blocking mAbs directed against CXCR4 or CXCL12, or control IgG (10µg ml^{-1}). Data are shown as mean±s.e.m (n=3). The statistical significance of differences between means of migrating cells (in %) in treated *versus* untreated cells was evaluated with Student's t-tests. The differences between the percentages of migrating cells towards Ad-CM isolated from mu-VAT from obese *versus* lean mice are also shown. **(c)** Inhibition of the CCR3/CCL7, but not CXCR4/CXCL12 axis totally abrogates the enhanced chemotaxis observed in obesity. The histograms represent the ratio of migration towards conditioned medium of primary isolated adipocytes from obese to lean mice in each treated conditions. Statistical analysis: * statistically significant by Student's t-test $p<0.05$, *** $p<0.001$, NS for not significant. n stands for the number of replicated independent experiments.

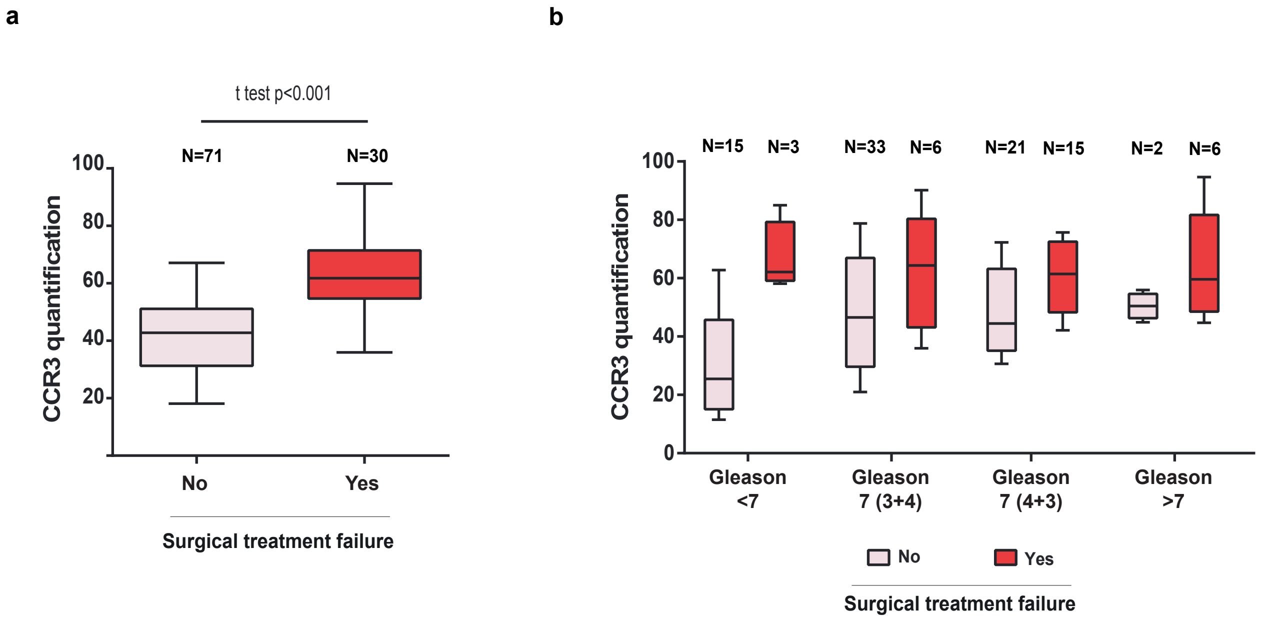

Supplementary Figure 12. CCR3 expression is higher in tumors from patients exhibiting surgical treatment failure in all classes of Gleason. (a) Boxplots of CCR3 values comparing patients with and without surgical treatment failure. We used Student's t-test to correlate CCR3 expression to the occurrence or not of a surgical treatment failure (which is a non-ordered variable represented by a category). The median values for CCR3 expression in the two categories are shown. **(b)** Boxplots of CCR3 values comparing patients at one year of follow-up within the four Gleason score classes. CCR3 values were systematically higher in patients with treatment failure regardless of Gleason score class. Discrepancies in CCR3 values tended to be greater in case of favorable Gleason score (<7). The boxes represent the median (black middle line) limited by the 25th (Q1) and 75th (Q3) percentiles. The whiskers are the upper and lower adjacent values, which are the most extreme values within Q3+1.5(Q3−Q1) and Q1−1.5(Q3−Q1), respectively. N defines the number of individuals within each group.

a

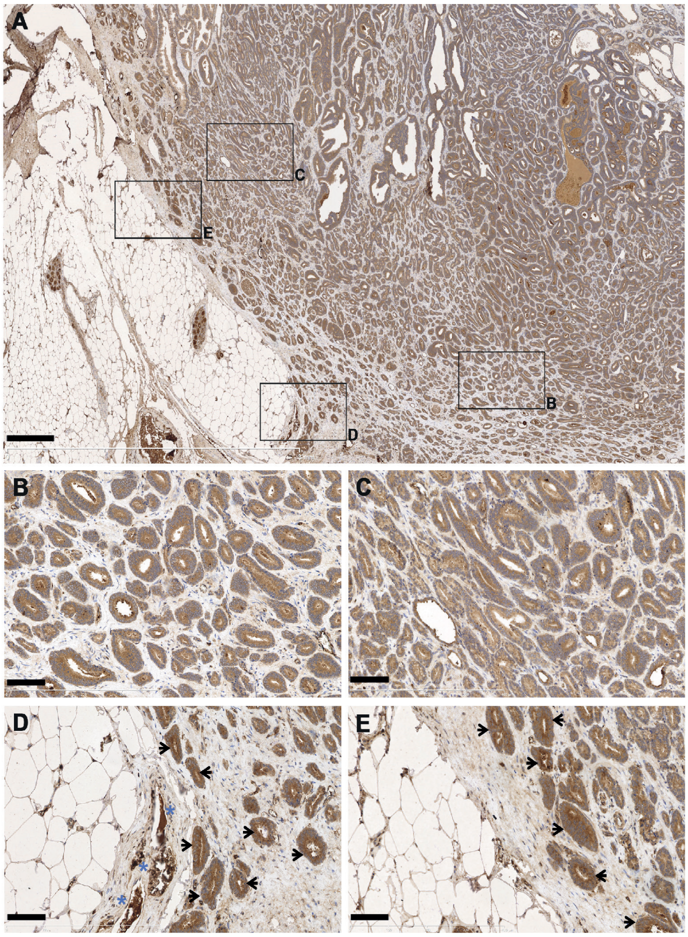

b

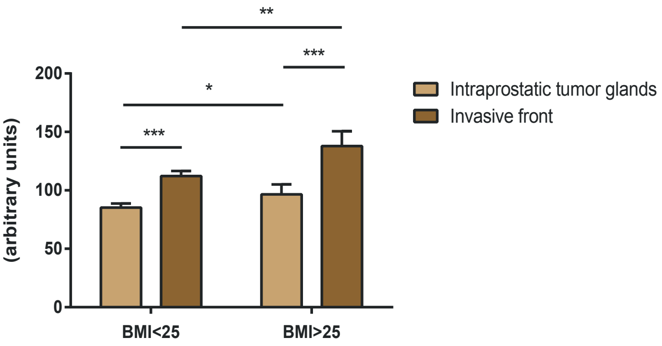

Supplementary Figure 13. CCR3 expression is increased in tumor cells present at the invasive front and this effect is increased in overweight and obese patients. (a) Immunohistochemical staining for CCR3 (with HE counter-staining) was performed in eight whole slices of human pT3a and pT3b prostatectomy pieces. Pictures show a representative experiment (obtained for patient with BMI = 28 kg m2^{-1}). A: picture of the whole slice after staining with CCR3; (B, C): zoom of the intra-tumoral CCR3 staining in two representative zones; (D, E): zoom of the CCR3 staining at the invasive front in two representative zones. Scale bars, for A, 500 µm and for B, C, D, E, 100 µm. **(b)** Histograms show CCR3 expression in tumor cells at the invasive front compared to intra-tumoral areas for four lean (BMI<25) and four overweight/obese patients (BMI>25). Data are shown as mean±s.e.m (n=4 tumors per group). Tumor glands (showed with black arrows) were defined with two pathologists and the stained areas of tumors were digitalized and protein expression was quantified using ImageJ software plugins. Note that some blood vessels (blue stars) or nerve tissues are also strongly stained but quantification only considered tumor glands. Statistical analysis: * statistically significant by Student's t-test p<0.05, ** p<0.01, *** p<0.001.

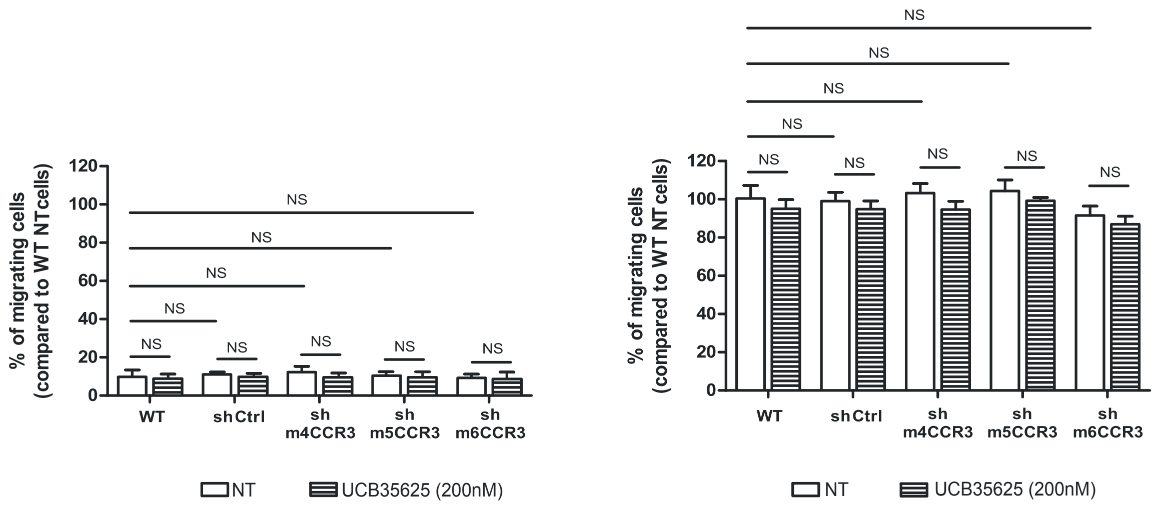

Supplementary Figure 14. CCR3 invalidation is without effect on prostate cancer cell migration when 10% FCS is used as chemoattractant. *In vitro* migration of the indicated cell lines towards 0% (left panel) or 10% FCS (righ panel) in the presence or absence of the CCR3 antagonist (UCB35625, 200nM). Note that the observed migration was similar between WT cells and cells transfected with control vector (shCtrl) or sh directed against CCR3. Furthermore, the migration was unaffected by treatment with the CCR3 antagonist whatever the cell lines considered. Data are shown as mean±s.e.m (n=3). Statistical analysis: NS for not significant. n stands for the number of replicated independent experiments.

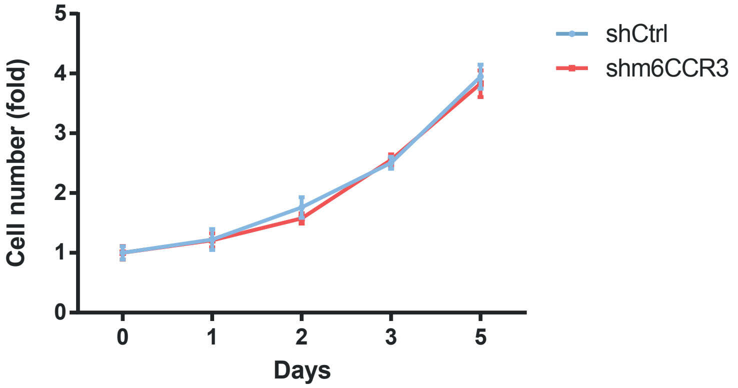

Supplementary Figure 15. Stable downregulation of CCR3 does not affect cell proliferation. $3x10^3$ TRAMP-C1P3 cells transfected with either control vector (shCtrl) or sh directed against CCR3 (shm6CCR3) were plated in quadruplicate for each time point in 96 well plates. When all the cells were attached after 6 h, the number of viable cells was measured by MTT assay and the mean optical density obtained was set at 1 (t0). Similar experiments were performed after 1, 2, 3 and 5 days and the values obtained were expressed as fold increase to control. Data are shown as mean±s.e.m (n=3). No statistically significant differences were observed between the two cell lines. n stands for the number of replicated independent experiments.

AC	MW (KDa)	Description	Gene Name	NE	Score	PN	% Coverage
IPI00116264	14408.9	C-X-C motif chemokine 5	Cxcl5	3	97.92	4	41
IPI00131236	11276.86	C-C motif chemokine 7	Ccl7	4	163.94	5	34
IPI00121856	10532.69	Growth-regulated alpha protein	Cxcl1	3	88.07	3	50
IPI00108087	16543.55	C-C motif chemokine 2	Ccl2	2	34.80	3	32
IPI00125187	14203.99	C-C motif chemokine 9	Ccl9	3	93.68	3	28
IPI00108061	10310.55	Isoform Alpha of Stromal cell-derived factor 1	Cxcl12	4	62.88	2	42
IPI00311383	26938.25	Adiponectin	Adipoq	4	250.76	6	26
IPI00120465	13110.37	Resistin	Retn	2	91.85	3	29

Supplementary Table 1: CCL7 chemokine is the only ligand of CCR3 secreted by mature adipocytes. Results from proteome analysis of Ad-CM (mature adipocytes 3T3-F442A); 4 independent experiments. Chemokines (highlighted in green) identified by nanoLC-MS/MS are listed. Results obtained for adiponectin and resistin, two well-known adipokines, are also indicated. The number of experiments in which proteins have been identified (NE), the best score (score), the number of peptides (PN) and the average sequence coverage for each protein are indicated. The presence of chemokines and adipokines in the conditioned medium was confirmed by ELISA and multiplex analysis. Data obtained (mean of two independent experiments) are as following: CCL2 (6.3ng ml^{-1} for $1x10^5$ cells), CCL7 (28.3ng ml^{-1} for $1x10^5$ cells), CXCL1 (32.6ng ml^{-1} for $1x10^5$ cells), CXCL12 (0.8ng ml^{-1} for $1x10^5$ cells), Adiponectin (32ng ml^{-1} for $1x10^5$ cells) and Resistin (1.7ng ml^{-1} for $1x10^5$ cells). Moreover seeing that CCL5 (absent of our proteomic analysis) has been identified in the secretion of mammary adipocytes we have determined CCL5 concentration in Ad-CM by ELISA. The results showed that the level of CCL5 was almost undetectable (mean concentration 0.01ng ml^{-1} for $1x10^5$ cells).

		CCR3			
		**low**	**moderate**	**high**	**p-value**
**Gleason score,** n (percentage per column)	Gleason <7	14 (66.7)	3 (11.1)	2 (4.6)	<0.001
	Gleason (3+4)	5 (23.7)	11 (40.7)	6 (14.0)	
	Gleason (4+3)	1 (4.8)	8 (29.7)	16 (37.2)	
	Gleason >7	1 (4.8)	5 (18.5)	19 (44.2)	

Supplementary Table 2: Immunohistochemical staining for CCR3, as well as hematoxylin counter-staining, was performed in normal epithelium (10 samples) and human prostate cancer tissues (91 tumors in duplicate). Two pathologists, who were blind to clinical data, independently scored CCR3 expression. The Gleason score characterizes the glandular architecture of the prostate based on a score that represents the level of cancer de-differentiation. The Gleason score is comprised of two numbers, each representing the most common Gleason patterns ranging from 1 to 5, where 1 represents a highly differentiated carcinoma and 5 represents an aggressive de-differentiated one. Therefore the highest Gleason score is 10 and the lowest 2. A related issue is the clinical usefulness of the percentage of high-grade carcinoma (Gleason patterns 4 and 5) in a tumor specimen. The correlation between CCR3 expression and Gleason score has been evaluated by Spearman rank correlation assuming a monotonic relation between considered variables.

		Variables	Percentage among the cohort
**Size of the cohort**		101	100
**Age at surgery (Years)**, median (extent)		63 (47-75)	
**Body mass index (BMI) (Kg/m²)**, median (extent)		26.1 (19-34)	
**PSA before surgery (ng/mL)**, median (extent)		7 (2-37)	
**Last PSA dosage (ng/mL)**, median (extent)		0 (0-4.02)	
**Prostatectomy piece weight (g)**, median (extent)		48 (22-93)	
**Length of the monitoring (Days)**, median (extent)		765 (62-1736)	
**Post-surgical treatment,** number of patients	Monitoring only	71	70.30
	Radiotherapy only	16	15.84
	Hormonotherapy only	7	6.93
	Radiotherapy and hormonotherapy	7	6.93
**Post-surgery evolution,** number of patients	Biochemical recurrence	14	13.86
	Surgical treatment failure [a]	30	29.70
	No relapse	71	70.30
	Death	1	0.99

Supplementary Table 3: All the patients included in the cohort underwent surgery for PCa between February 1st 2010 and December 1st 2011 at the Urology Department of Toulouse Hospital. Surgical treatment consisted of a robot-assisted radical prostatectomy, associated in some cases with lymphadenectomy (standard or extended). All patients included in the study had localized disease without metastasis at the time of the surgery. Biochemical recurrence was defined by two PSA readings >0.2ng ml^{-1} according to the European Association of Urology guidelines. [a] defined by patients that exhibit either biochemical recurrence, locoregional recurrence or distant metastases, or by use of adjuvant radiation or hormonal deprivation therapy. Abbreviations used: BMI, Body Mass Index; PSA, Prostate Specific Antigen.

		Variables	Percentage among the cohort
**Gleason score,** number of patients	<7	18	17.82
	=7 (3+4)	39	38.62
	=7 (4+3)	36	35.64
	>7	8	7.92
**Percentage of low differentiated contingent (grade 4 and 5)**, median (extent)		40 (0-100)	
**Tumor localization**, number of patients	Transition zone	10	9.90
	Peripheral zone	91	90.10
**pT stage**, number of patients	pT2b	6	5.94
	pT2c	35	34.65
	pT3a	44	42.57
	pT3b	16	15.84
**Positive surgical margins**, number of patients		22	21.78
**Lymphatic emboli**, number of patients	No	93	92.08
	Yes	8	7.92
**Bilateral lymphadenectomy**, number of patients		79	79.21
**Lymph node invasion**, number of patients	Nx (No node dissection performed)	22	21.78
	N0	74	73.27
	N1	5	4.95
**CCR3 expression (arbitrary units)**, median (extent)		48.5 (11.4-94.7)	

Supplementary Table 4: Histological and immunohistochemical characteristics of the cohort. The Gleason score has been defined in Supplementary Table 2. The pT staging represents the size of the tumor as determined from the prostatectomy specimen. In pT2 tumors, the tumor is confined to the prostate gland; pT2b tumors involve more than one half of a lobe and pT2c tumors involve both lobes, For pT3, the tumor extends through the prostate capsule; pT3a tumors exhibit unilateral or bilateral extension and pT3b tumors invade seminal vesicles.
